# Insight into Mantle Cell Lymphoma Pathobiology, Diagnosis, and Treatment Using Network-Based and Drug-Repurposing Approaches

**DOI:** 10.3390/ijms25137298

**Published:** 2024-07-02

**Authors:** Georgia Orfanoudaki, Konstantina Psatha, Michalis Aivaliotis

**Affiliations:** 1 Functional Proteomics and Systems Biology (FunPATh), Center for Interdisciplinary Research and Innovation (CIRI-AUTH), Balkan Center, GR-54124 Thessaloniki, Greece; orfanoudaki@gmail.com (G.O.); kpsatha@auth.gr (K.P.); 2 Institute of Molecular Biology and Biotechnology Foundation for Research and Technology—Hellas, GR-70013 Heraklion, Greece; 3 Laboratory of Medical Biology—Genetics, School of Medicine, Faculty of Health Sciences, Aristotle University of Thessaloniki, GR-54124 Thessaloniki, Greece; 4 Basic and Translational Research Unit, Special Unit for Biomedical Research and Education, School of Medicine, Aristotle University of Thessaloniki, GR-54124 Thessaloniki, Greece; 5 Laboratory of Biological Chemistry, School of Medicine, Aristotle University of Thessaloniki, GR-54124 Thessaloniki, Greece

**Keywords:** mantle cell lymphoma, non-Hodgkin lymphoma, differentially expressed, stages, biomarkers, drug repurposing

## Abstract

Mantle cell lymphoma (MCL) is a rare, incurable, and aggressive B-cell non-Hodgkin lymphoma (NHL). Early MCL diagnosis and treatment is critical and puzzling due to inter/intra-tumoral heterogeneity and limited understanding of the underlying molecular mechanisms. We developed and applied a multifaceted analysis of selected publicly available transcriptomic data of well-defined MCL stages, integrating network-based methods for pathway enrichment analysis, co-expression module alignment, drug repurposing, and prediction of effective drug combinations. We demonstrate the “butterfly effect” emerging from a small set of initially differentially expressed genes, rapidly expanding into numerous deregulated cellular processes, signaling pathways, and core machineries as MCL becomes aggressive. We explore pathogenicity-related signaling circuits by detecting common co-expression modules in MCL stages, pointing out, among others, the role of VEGFA and SPARC proteins in MCL progression and recommend further study of precise drug combinations. Our findings highlight the benefit that can be leveraged by such an approach for better understanding pathobiology and identifying high-priority novel diagnostic and prognostic biomarkers, drug targets, and efficacious combination therapies against MCL that should be further validated for their clinical impact.

## 1. Introduction

MCL is a rare, aggressive, heterogeneous subtype of B-cell lymphoproliferative neoplasia that comprises about 7% of all adult-onset NHLs [1,2]. An ICC/WHO 2022 update classified MCL into two categories: nodal or conventional MCL (hereon in classical cMCL) (80–90% of cases) and non-nodal leukemic MCL (nnMCL) (10–20% of cases) [1,2]. cMCLs are characterized mainly by unmutated IGHV, mutated TP53, high levels of oncogenic SOX11, and aggressive behavior. NnMCLs bear mutant IGHV, fewer genetic alterations, are SOX11-negative (or weakly express it), and clinically indolent; however, adverse prognosis can be conferred by TP53 alterations and genomic complexity [1,2]. Histologically, pleomorphic and blastoid morphology variations can be distinguished from cMCL, with blastoid MCL often exhibiting a high Ki67 proliferative index, resulting in a more aggressive clinical outcome [1,2,3].

The majority of MCL cases (>95%) feature the abnormal chromosomal translocation t(11;14)(q13;q32)(IgH/CCND1), leading to constitutive cyclin D1 (CCND1) overexpression inducing cell-cycle deregulation, along with deregulation of DNA damage and repair mechanisms and transcriptional alterations [3,4,5]. However, overexpressed CCND1 is not always pathognomonic for MCL initiation, development, and progression (CCND2, CCND3, CCNE translocations may alternatively deregulate cell cycle progression) [4], and comprehensive profiling at the genome, epigenome, transcriptome, and proteome level is often necessitated, although still far from clinical practice [3,6,7]. The frequently occurring relapses after initial treatment with increasing resistance to chemotherapy make MCL the B-cell lymphoma with the worst clinical outcome, [3,8,9] while only a very small percentage of cases show an indolent clinical course [9]. Therefore, it is critical to define the various molecular subgroups of MCL to investigate putative risk-adjusted therapy strategies.

Presently, high-throughput transcriptomic and proteomic analyses have been instrumental in revealing constitutively activated and/or deregulated proteins and pathways in MCL [10,11,12], as well as potential prognostic factors [13,14,15]. In this context, the advent of drug repurposing (DR) as a worthwhile therapeutic strategy delivering “second chances” for the reappraisal of specific disease-oriented and molecular-targeted drugs, opens avenues of enhanced opportunities for MCL patients towards the systematic improvement of health outcomes and health-care costs [16,17]. However, despite best efforts, intuitive computational approaches that piece together the jigsaw puzzle-like information contained in such publicly available big data are still lacking. Our approach leverages a promising integrative bioinformatic pipeline aimed at understanding MCL pathobiology through network-based methods and drug-repurposing strategies to uncover drug targets and propose drugs that might be successfully repurposed against MCL.

## 2. Results

**Figure 1:**
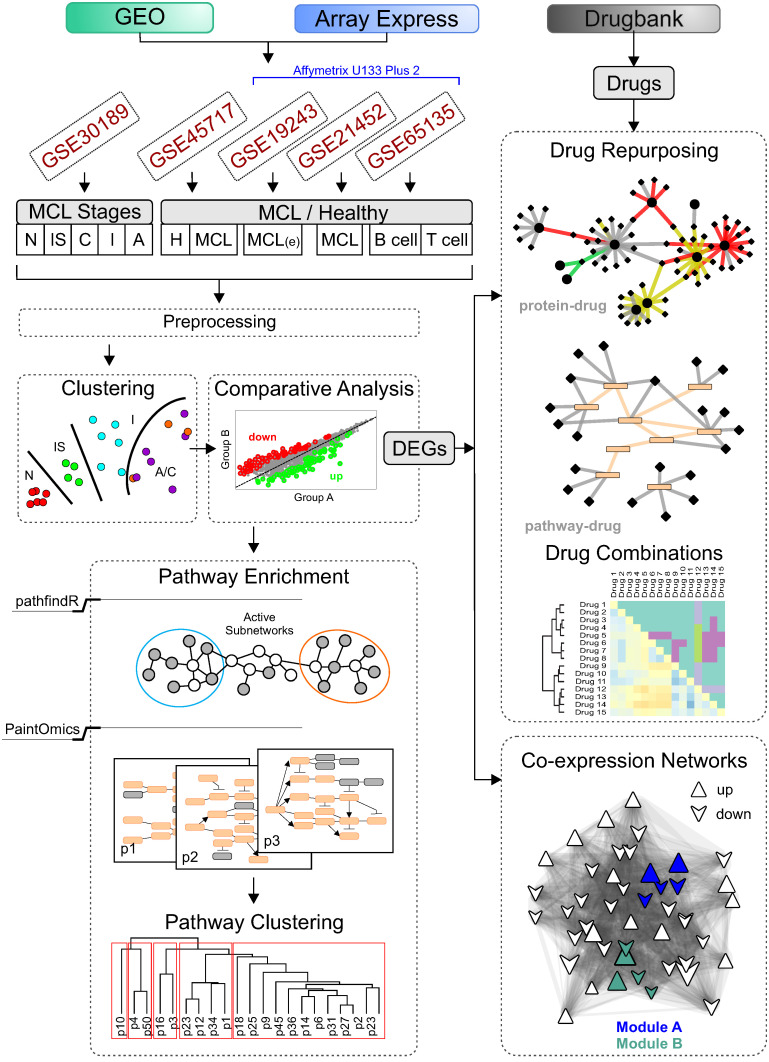
Computational analysis pipeline. Transcriptional data obtained measuring RNA levels of tissue or blood samples of MCL patients and normal cases following a modular pipeline of network-based methods. Five microarray studies were revisited. Kimura et al. (GSE30189) isolated tissue samples of 17 CCND1-positive MCL patients at four stepwise distinct stages (in situ, classical, intermediate and aggressive) and four normal mantle zone B lymphocytes samples. Espinet et al. (GSE45717) collected PB B cells of five typical MCL patients and eight healthy individuals and obtained transcriptional data using Affymetrix Human Exon 1.0 ST arrays. Three Affymetrix Human U133 Plus 2 datasets were also analyzed. Leshchenko et al. (GSE19243) purified CD19+ fractions from peripheral blood of newly diagnosed MCL patients (five selected). Hartmann et al. extracted RNA from lymph node specimens (GSE21452) of 64 previously untreated MCL patients. Finally, Newman et al. (GSE65135) compared different human hematopoietic cell phenotypes, including B cells of five healthy tonsils. First, transcriptional data were preprocessed, then comparative analysis was performed between pairs of different sample groups. SVM models were also trained using the most variable genes of each sample group, and MCL and samples were recategorized accordingly. The Limma R package was employed and differentially expressed genes (DEGs) were selected based on an adjusted *p*-value threshold of 0.05. Next, DEGs were used as an input to four independent subsequent analysis: pathway enrichment (performed by two bioinformatic tools pathfindR [18] and PaintOmics [19]), drug repurposing, co-expression network spectral clustering, and compound drug combination prediction analyses.

Publicly available comparative transcriptomic data on mRNA relative abundance in tissue and blood samples of MCL patients and normal individuals were carefully selected and reanalyzed following a modular pipeline of network-based methods (Figure 1). Five microarray studies were revisited, as described in the Section 4. In the following paragraphs, the findings of this analysis are described in detail.

### 2.1. Multidimensional Scaling Analysis and Hierarchical Clustering Uncovers Distinctive Profiles in MCL vs. Healthy Donors and Increased Heterogeneity in MCL Stages

Multidimensional scaling (MDS) analysis of the 500 most variable transcripts shows that normal (benign lymphadenitis, BL) and the in situ MCL stage (InS) are well separated considering the leading dimensions of the analysis (Supplementary Figure S1). On the contrary, the classical stage (cMCL) seems to overlap with the aggressive and intermediate stages (Supplementary Figure S1; intMCL, agrMCL). RNA extracted from the peripheral blood (PB) B cells of five typical MCL patients and eight healthy individuals (MCL1-n, H1-n samples; Supplementary Figure S2) suggests that MCL transcriptional profiles are more disparate compared to the healthy ones, implying lymphoma heterogeneity. The dispersion of MCL cases is even more pronounced when more MCL cases are included in the MDS analysis (Supplementary Figures S3B and S2). Newly diagnosed MCL patients exhibit higher similarity based on the five analyzed cases (samples in cyan, Supplementary Figure S3B).

Hierarchical clustering heatmaps of the 200 most variable genes are in accordance with the MDS analysis results. Clustering of the MCL stepwise stages generally agrees with the original labeling of the samples (Supplementary Figure S4A); normal, in situ, and intermediate samples are grouped together, whereas classical stage samples are similar to the average profile of the aggressive group (Supplementary Figure S4A). Typical MCL cases exhibit distinct profiles compared to the healthy ones (Supplementary Figure S4B). Notably, batch effect correction reveals distinct clustering of patients’ transcription profiles, fitting well with the five newly diagnosed patients (Supplementary Figure S3A).

### 2.2. Comparative Transcriptomic Analysis Reveals Significant MCL Stage-Dependent Deregulation of Gene Transcription

**Figure 2:**
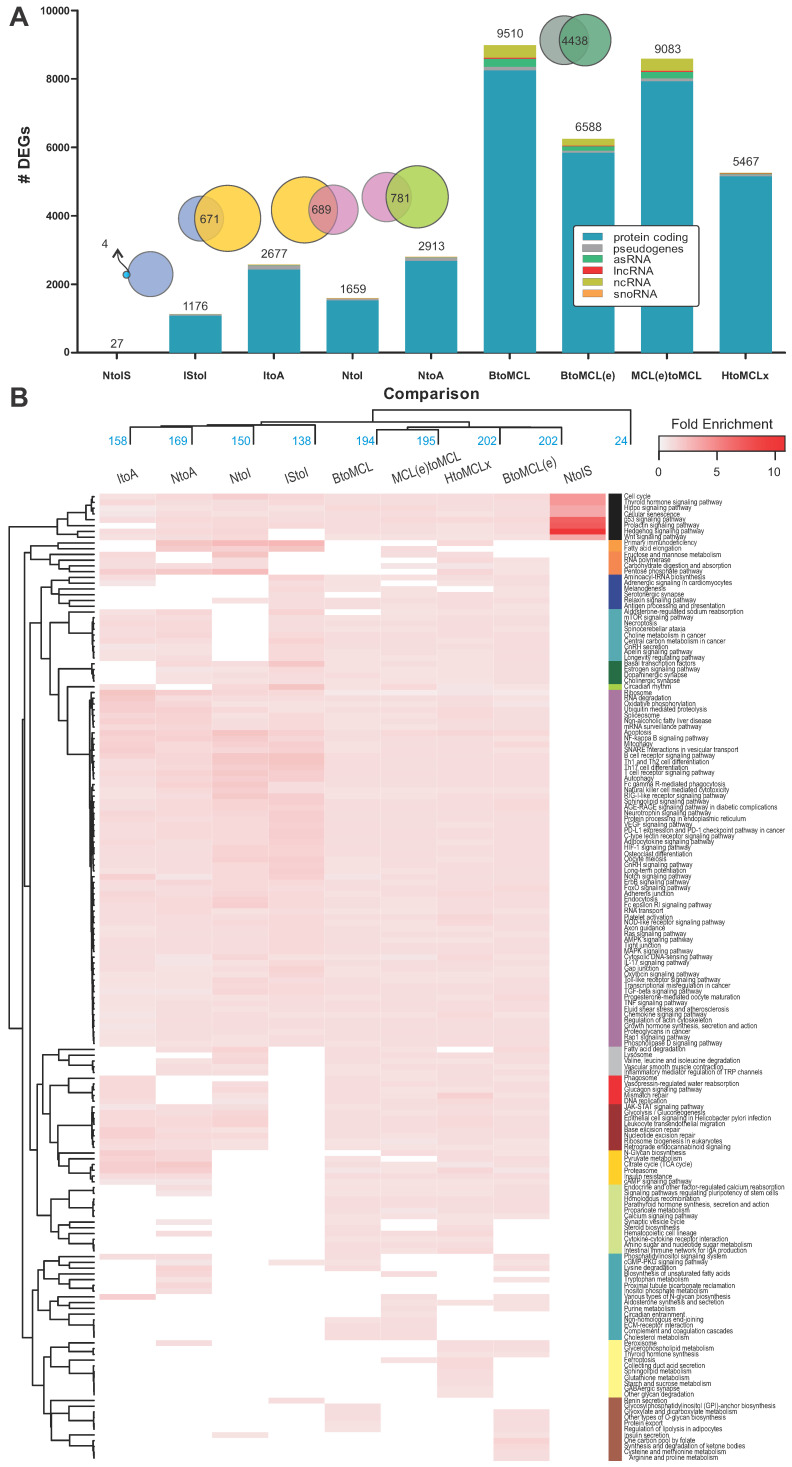
Differentially expressed genes and enriched biological pathways at different MCL stages. (**A**) Bar chart summarizing the number of identified DEGs at each stepwise MCL stage comparison, including normal (N) to any other MCL stage transitions. Results of the comparative analysis between healthy and typical MCL patients (HtoMCLx), normal B cells and newly diagnosed MCL patients (BtoMCL(e)), normal B cells and progressed-MCL-stage patients (BtoMCL), and newly diagnosed and progressed-MCL-stage patients (MCL(e)toMCL) are also depicted. Differentially expressed transcripts are also colored based on their type (blue: protein coding, gray: pseudogenes, green: asRNAs, red: lncRNAs, yellow: ncRNAs and orange: snoRNA). The overlap between the different sets of DEGs is represented in the form of Venn diagrams at the top of the bar chart. (**B**) Heatmap of 168 enriched KEGG pathways based on the *FE* calculated by the PathfindR [18] bioinformatic tool for the different sample group comparisons (Table 2). Enriched pathways (rows) and sample comparisons (columns) were hierarchically clustered. For limited space reasons, disease-specific pathways are not depicted (e.g., “bladder cancer” or “thyroid cancer”).

Comparative transcriptomics between sample groups of merged MCL stages vs. healthy controls, as well as stepwise MCL stage comparisons, revealed significant differences in the transcription levels of several genes (Table 1 and Table 2; Figure 2A). The detected differences are both qualitative and quantitative, with a pronounced increase in the number of DEGs as MCL progresses to an aggressive stage (Figure 2A, Supplementary Table S4). Progression from normal mantle zone B cells to the in situ MCL phenotype (NtoIS; Supplementary Table S2) comprises a “precursor” set of 27 DEGs. Transition to the subsequent stages leads to a more pronounced increase in the number of DEGs, which are doubled at the final stage (1176 and 2677; IStoI and ItoA, respectively; Figure 2A). Four genes, corresponding to zinc finger protein 219, tumor necrosis factor ligand 4, cannabinoid receptor 1, and B- and T-lymphocyte attenuator, are common between NtoIS and IStoI stage transitions (Figure 2A). Different DEGs seem to be involved in the intermediate and aggressive stage progressions, indicated by their overlap (671 common DEGs; ~52%; Figure 2A). On the other hand, a substantial number of 5467 DEGs was identified when PB B cells of five typical MCL patients were compared against eight healthy ones (Figure 2A; HtoMCLx). Interestingly, the number significantly increased to 9510 DEGs when the number of patient cases was increased (BtoMCL; Figure 2A), possibly emphasizing each patient’s unique contributions to cellular and molecular heterogeneity.

### 2.3. Linear Increase in Deregulated Biological Pathways as MCL Progresses to the Aggressive Form

PathfindR [18] and PaintOmics [19] were employed for the identification of enriched terms in different sample group comparisons (Table 2, Figure 1 and Figure S5). There are qualitative and quantitative differences between the two tools, which can be attributed to the different algorithmic approaches, the type of identifier used (gene symbol, ensemble ID), or some information loss after transcript identifier mapping. A cluster heatmap of the fold enrichment values (*FE*) summarizes the enriched KEGG pathways identified by pathfindR in all nine sample group comparisons (Figure 2B, Supplementary Table S5). Progression from the normal B-cell to the in situ MCL profile (NtoIS), showed a distinct pathway enrichment phenotype, involving less pathways compared to more progressed stages (IStoI, ItoA, Table 2, Figure 2B) but with a more intense *FE*. At the onset of MCL, pathfindR identified 24 significantly enriched pathways and PaintOmics only 2 (Table 2, Supplementary Table S5). “Hedgehog signaling pathway” was identified by both tools and “microRNAs in cancer” was pointed out only by PaintOmics (Supplementary Table S5A vs. B). A total of 16 out of 24 pathways were disease-specific biological networks (e.g., “breast cancer” or “melanoma” pathways, Supplementary Table S5A); the remaining eight deregulated pathways were “p53” and “Wnt” signaling pathways, “cell cycle”, “cellular senescence”, “Hedgehog”/“prolactin”/“thyroid hormone”/“Hippo” signaling pathways (Figure 2B, Supplementary Table S5A). The common denominator in all paths was the upregulated G1/S-specific cyclin D1 gene (Supplementary Table S5).

Uniquely identified pathways in each of the stepwise MCL stage comparisons outline the course of the disease. Progression to intermediate stage (IStoI) was characterized by the deregulation of “MAPK”, “VEGF”, “mTOR”, “TNF”, “NF-kappa B”, “BCR” signaling pathways, “Gap”/“Tight”/“Adherens junction synapses” and core complexes and organelles, as well as “splicesome”, “ribosome”, “RNA polymerase”, and “lysosome” (Figure 2B, Supplementary Table S5A,C). The subsequent ItoA transition was characterized by the deregulation of “proteasome”, “phagosome”, and “peroxisome”, metabolic pathways like “pyruvate metabolism”, “citrate cycle (TCA cycle)”, “N-glycan biosynthesis”, “lysine degradation”, “cAMP signaling pathway”, and other pathways, such as “endocrine and other factor-regulated calcium reabsorption”. When intermediate and aggressive stages were directly compared to the normal state (NtoI, NtoA), pathfindR identified significantly more enriched pathways (150, 169, respectively) than PaintOmics (39, 21, respectively; Table 2). Sixteen and seven KEGG pathways were commonly found between the tools for IStoI and ItoA comparisons. Among them were “Th1 and Th2 cell differentiation”, “Th17 cell differentiation”, “Ras signaling pathway”, “estrogen signaling pathway”, “ribosome”, “oxidative phosphorylation”, “RNA degradation”, “retrograde endocannabinoid signaling” and “glucagon signaling pathway”. The analysis of additional data of MCL patients and healthy donors (Affymetrix Human Genome U133—Table 1), contributed extra enriched pathways, such as “calcium signaling pathway”, “cytokine–cytokine receptor interaction”, “steroid biosynthesis”, “circadian entrainment”, and “synthesis and degradation of ketone bodies” (Supplementary Table S5A).

Pathways related to pathogen and viral infections were also found: “shigellosis”, “Salmonella infection”, “Yersinia infection”, “toxoplasmosis”, “leishmaniasis”, “Epstein–Barr virus infection”, “human T-cell leukemia virus 1 infection”, “influenza A”, and more. Signaling pathways described in other types of cancer are also included in the list of enriched pathways, such as “breast”, “non-small cell”, “thyroid”, “bladder”, “endometrial”, “pancreatic” and “colorectal” cancer.

Reactome, BioCarta and GO enriched terms were also identified by pathfindR (Supplementary Table S5D–F) for the nine sample comparisons performed in the current analysis (Table 2). PathfindR enrichment analysis of Reactome terms uncovered more specific cellular processes, such as “SUMOylation”-, “RUNX1”-, and “Rho GTPase cycle”-related pathways or “TNFR2 non-canonical NF-kB pathway”, “downregulation of SMAD2/3:SMAD4 transcriptional activity”, and “Ras activation upon Ca^2+^ influx through NMDA receptor”.

Seven, five, and three, Reactome, BioCarta and GO terms, respectively, were enriched in the in situ MCL stage (NtoIS; Supplementary Table S5D–F). Among the Reactome terms were “transcriptional activity of SMAD2/SMAD3:SMAD4 heterotrimer”, “ubiquitin-dependent degradation of cyclin D”, “transcriptional regulation by VENTX” and “pre-NOTCH transcription and translation”. “BTG2”, “P53”, “WNT”, “CARM_ER” and “G1” BioCarta pathways and “protein tyrosine kinase binding”, extracellular matrix”, and “T cell co-stimulation” GO terms were also pointed out. Seventy-four unique Reactome terms were selected in IStoI comparison, including “MAP2K and MAPK activation”, “TRAF6-mediated IRF7 activation”, “CTLA4-inhibitory signaling”, “signaling by Hippo” and “signaling by WNT in cancer”. Unique Reactome terms in the ItoA stage transition included “MAPK6/MAPK4 signaling”, “estrogen-dependent gene expression”, “signaling by NOTCH”, “p53-dependent G1 DNA damage response”, “SUMOylation of DNA damage response and repair proteins”, “pexophagy”, “chromosome maintenance”, and “signaling by NOTCH”.

We constructed a pathway–pathway network of enriched KEGG pathways in all three stepwise MCL stage comparisons (NtoIS, IStoI, ItoA; Supplementary Figure S8A–C), where the thickness of the edges and the size of the nodes is proportional to the number of shared genes and the *FE*, respectively. NtoIS transition included eight pathways, all sharing the cyclin D gene (Supplementary Figure S8A). In the intermediate and aggressive stages, additional pathways under the scope of cyclin D were “triggered”, including “AMPK”, “JAK-STAT”, “FoxO”, “apelin”, “oxotocin” and “AGE-RAGE” signaling pathways and “tight junction” (Supplementary Figure S8B,C).

### 2.4. Spectral Clustering Algorithm Detects Common Modules between Gene Co-Expression Networks

PE analysis employs public repositories that organize established molecular networks, and thus is limited by the current state of knowledge of biological processes involved mainly in physiological conditions. To explore novel MCL-related gene correlations, we constructed co-expression networks of different sample groups (including MCL stages) using the MRNETB algorithm. Networks were compared for common modules using the spectral clustering approach described by Zhang et al. [23,24]. Corresponding sample comparisons were made (Table 1), and the number of modules was decided based on the respective eigengaps (Supplementary Figure S7, Supplementary Table S7).

**Figure 3:**
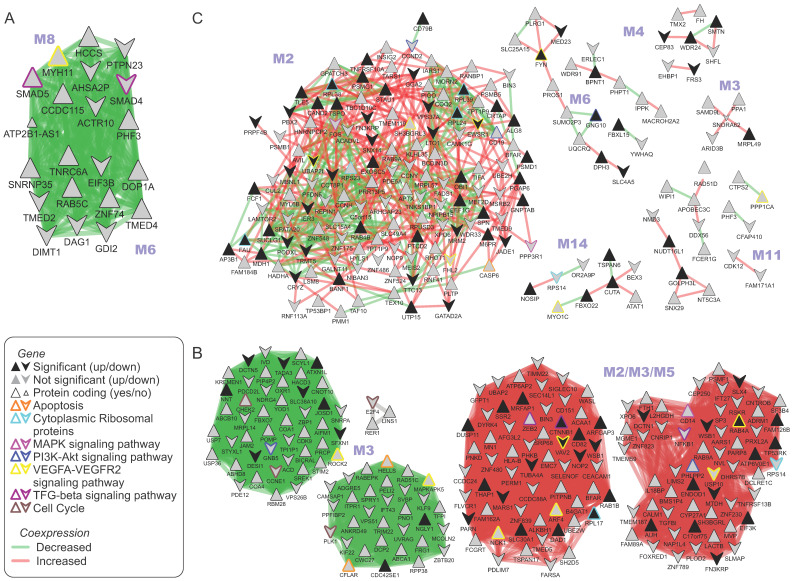
Co-expression network spectral clustering. Gene co-expression networks at different MCL stages were constructed using the MRNETB algorithm [25]. Modules of co-expressed genes were identified and aligned between networks using a spectral clustering approximation approach [23,24]. The union of the aligned modules between (**A**) normal and in situ, (**B**) in situ and intermediate, (**C**) intermediate and aggressive are depicted. Up- and down-regulated genes are depicted as regular or flipped triangles, respectively. The log_2_ fold change of the co-expression between MCL stages is used as a weight of the edges. Loss of co-expression is depicted with green (negative log_2_ fold change), whereas gain of co-expression with red (positive log_2_ fold change). As a result, “green communities” are genes where co-expression relationships are weakened as the MCL progresses to the next stage. The node border line color depicts the Wiki pathway in which a gene participates. A gene can participate in more than one pathway, but here we show only selected pathways (orange = apoptosis, cyan = cytoplasmic ribosomal proteins, magenta = MAPK signaling pathway, blue = PI3K–Akt signaling pathway, yellow = VEGFA–VEGFR2 signaling pathway, brown = cell cycle, purple = TGF-beta signaling pathway).

Two common modules were found between the normal and in situ sample groups (Figure 3A). Worth mentioning are the genes: asRNA ATP2B1-AS1 and myosin heavy chain 11 (MYH11), both members of the “VEGFA–VEGFR2 signaling pathway” (Figure 3A). GSEA analysis of the modules identified five wiki pathways, including “TGF-beta receptor signaling” with genes SMAD4/5 (Supplementary Table S7A).

Comparing in situ and intermediate stages required significant parameter calibration (q threshold, beta parameter) and common modules were not easily identified. We provide two alternative different parameter configurations (Supplementary Table S7A; Figure 3B).

Six aligned modules were discovered between intermediate and aggressive co-expression networks (Figure 3C). GSEA analysis pointed out the prevalence in cytoplasmic ribosomal proteins and eleven genes of the VEGFA–VEGFR2 signaling pathway (Supplementary Table S7).

Lastly, the common modules between normal B cells and newly diagnosed MCL and advanced MLC patients are summarized (Supplementary Figure S8). GSEA analysis for the common module in aggressive MCL revealed genes related to miR-509-3p and focal adhesion (VEGFA, COL5A1, HSP90AA1, ITGB6, PFKFB2) [25]. Interestingly, two non-coding RNAs (LINC00112, LINC01003) and two antisense RNAs, (UNC5B-AS1 and IGF2BP2-AS1) participate in the module for aggressive MCL (Supplementary Figure S8A).

The module identified in early MCL cases highlights folate metabolism genes (INSR, SLC46A1 and AHCY; Supplementary Figure S8B). Finally, three common functional modules between early and advanced MCL were discovered (Supplementary Figure S8C). Among the significantly up- or down-regulated ones are six non-protein-coding RNAs, two antisense RNAs (LBX2-AS1 and SNTG2-AS1), four ncRNAs (LINC01139, LINC01618, LINC01706) and the long non-coding oncogene SNHG7. GSEA pointed out 37 genes of the VEGFA–VEGFR2 signaling pathway (e.g., JUN, STAT1, CLIC1, FYN, ACT1) and seven genes of the cytokine or interleukin signaling pathways (Supplementary Table S7A).

### 2.5. Drug Repurposing Recommends New Potential MCL Stage-Specific Treatments

**Figure 4:**
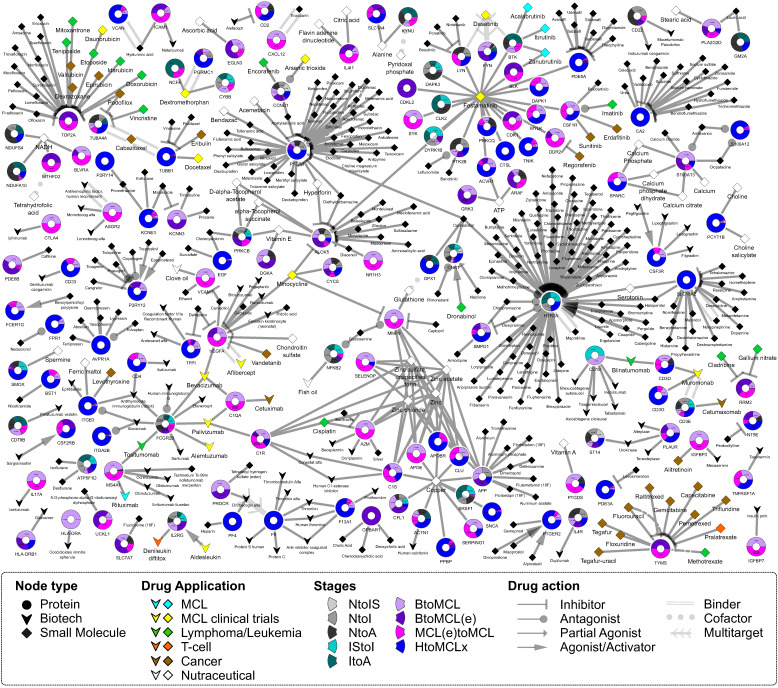
Proposed drugs for the distinct stages of MCL. Bipartite drug–gene graph of the 388 FDA-approved drugs proposed in the current analysis for the treatment of MCL. The potential drugs were obtained using DrugBank’s [26] catalogue of FDA-approved drugs and their gene/protein targets. Drug–gene pairs were selected based on the top DEGs identified in nine sample group comparisons (Table 1) and filtered given the type of action. Genes and drugs are depicted as circles and diamonds/triangles, respectively. Two types of drugs exist in the DrugBank database [26], the small molecules (diamonds) and the biotech molecular entities (arrowheads). Drugs are colored based on their original indication or clinical trial: MCL drugs (cyan), T-cell lymphomas (orange), other lymphoma/leukemia subtypes (green), drugs at clinical trial for the treatment of MCL [28] (yellow, drugs indicated in other types of cancer (brown) and nutraceutical substances (white). Each gene circle is a donut chart, where each donut slice represents one of the nine sample group comparisons where the gene was found to be significantly deregulated. Donut slices (i.e., sample comparisons) have the following color code: comparisons of the three MCL stages to the normal case (NtoIS, NtoI, NtoA) are gray-shaded with darker shades corresponding to more progressed MCL stage; light and dark teal represent IStoI and ItoA transitions; light and dark purple correspond to BtoMCL and BtoMCL(e) comparisons, whereas magenta corresponds to MCL(e)toMCL and blue to HtoMCLx. Finally, drug–gene relationships are characterized by the type of drug action (inhibition, agonist, antagonist etc.), and are represented either with different target arrow shapes or line types.

**Figure 5:**
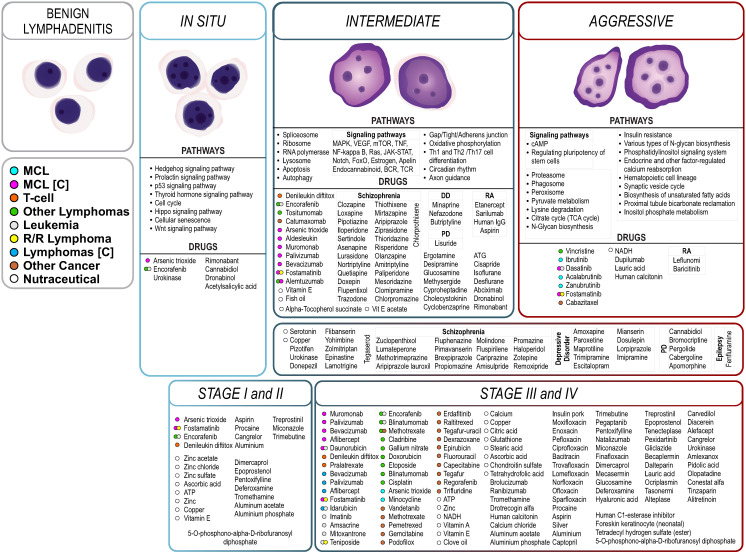
Synopsis of stage-specific pathways and the proposed MCL treatments, summarizing biological pathways that participate in MCL progression along with proposed treatments, from benign lymphadenitis to in situ, intermediate, and finally aggressive stage. Proposed drug treatments are colored or grouped based on their previous indication(s) or their clinical trial state regarding MCL or lymphoma treatment ([C]: at clinical trial). As proof of our approach, a considerable number of compounds are indicated in MCL (cyan), T-cell lymphoma (orange), other lymphomas (green), leukemia (gray), R/R lymphoma, or other cancer treatments. Some of them are at clinical trials for the treatment of MCL (magenta) or other subtypes of lymphoma (blue). Our approach also identifies groups of compounds originally prescribed in other diseases such as schizophrenia or bipolar disease (DD: depressive disorder; RA: rheumatoid arthritis or osteoarthritis; PD: Parkinson’s disease).

Following the identification of individual genes that were differentially transcribed and the corresponding pathways that were enriched, we applied a DR approach that proposes stage-specific FDA-approved drugs (Figure 4 and Figure 5). A drug–gene network was constructed given the drug–target relationships catalogued in DrugBank [26]. We considered as new potential treatments the FDA-approved drugs with a “targeting” action upon at least one of the top DEGs. Additional criteria that consider both the type of action (inhibition, antagonist, etc.) and the mode of change (up/down) were applied (Figure 4, Figure 5 and Supplementary Figure S5; Supplementary Methods). In total, 388 FDA-approved drugs are targeting the protein products of the top DEGs (Supplementary Table S6, Figure 4). As proof of concept of our approach, several compounds currently used in the therapy of MCL are among them (Figure 4; diamonds in cyan). In sum, 3 out of the 388 recommended drugs are currently used in MCL (ibrutinib, acalabrutinib, zanubrutinib), 13 and 8 are at clinical trials of MCL and nHL cases, respectively, 2 are administered in T-cell lymphoma patients, 4 in lymphoma in general, and 9 in leukemia (Supplementary Figure S9A). Interestingly, 59 out of the 388 drugs (~18.6%) are used for the treatment of mental illnesses: schizophrenia, psychotic disorders, depressive disorder, bipolar disorder, and ADHD (Supplementary Figure S9B). Fifty-five of these drugs target the serotonin receptor (HTR2A), and three drugs target two proteins: carbonic anhydrase CA2 and synaptic vesicular amine transporter SLC18A2. Other indications of the proposed drugs include breast, colorectal, ovarian, and bowel cancer, and other diseases, such as rheumatoid arthritis, osteoarthritis, and Parkinson’s disease (Supplementary Figure S9B). Twenty-three of the molecular entities are dietary supplements, thirty have anti-inflammatory use, nine are analgesic and thirteen are antibiotic agents. Moreover, based on DrugBank’s categorization, 31% of the proposed drugs are organoheterocyclic compounds and 18% are nutraceutical, whereas most of them are small molecules or organic compounds (Supplementary Figure S9C,D).

At the initiation of MCL, fewer DEGs are identified, leading to fewer potential drugs discovered (Figure 2A and Figure 4). Arsenic trioxide, an antagonist, and encorafenib, an inhibitor of CCND1 protein, are proposed at the in-situ stage; other compounds discovered are cannabidiol, rimonabant and dronabinol for targeting the overexpressed cannabinoid receptor 1 (CNR1). Cannabidiol is a negative allosteric modulator, rimonabant a potent and selective cannabinoid CB1 receptor antagonist, and dronabinol stimulates both CB1 and CB2 cannabinoid receptors.

More potential protein targets are found at the aggressive stage of MCL, leading to a greater variety of new drugs and targeted biological pathways (Figure 4). Seventy-five of the proposed MCL drugs target HTR2A, forty prostaglandin G/H synthase 1 (PTGS1), twenty-one DNA topoisomerase 2-alpha (TOP2A), fourteen amyloid-beta precursor protein (APP), fourteen carbonic anhydrase 2 (CA2) and thirteen vascular endothelial growth factor (VEGFA).

In sum, 116 of the proposed drugs target proteins at the two final stages of MCL (Figure 4). Interestingly, among them are metabolites (oxygen, NADH, ATP, serotonin molecules), and vitamins (vitamin E and derivatives, vitamin A) or zinc used as nutrition supplements. In total, 21 metabolites and nutrients were proposed as “drugs” (Figure 4, Figure 5, Supplementary Table S6).

Fostamatinib, a compound of interest, is currently in clinical trials for the treatment of lymphoma (including MCL) and leukemia. Fostamatinib is a prodrug converted to its active metabolite R406 in the intestine. R406 is a tyrosine kinase inhibitor exhibiting activity against spleen tyrosine kinase SYK, a key player of the BCR pathway [27,28] (Table S1). SYK was found to be overexpressed in the 63 MCL patient samples versus normal B cells. Additional potential targets of R406 are Bruton tyrosine kinase (BTK) and other tyrosine kinases, such as FYN, CSF1R and PTK2B [28].

Aldesleukin (non-glycosylated human interleukin 2), produced using genetically engineered *E. coli* strains, is documented as an agonist of the IL2RG cytokine receptor and is proposed at the intermediate stage (Figure 4; Supplementary Table S6). Aldesleukin in combination with cyclophosphamide, fludarabine or immunotherapy is currently clinically being tested in treating R/R MCL or other types of nHL (Table S1, trial NCT00924326). Denileukin diftitox is a recombinant DNA-derived immunotoxin administered against malignancies expressing the IL-2 receptor (e.g., cutaneous T-cell lymphoma).

Aflibercept, bevacizumab and minocycline are three out of thirteen compounds targeting vascular endothelial growth factor (VEGFA), which is active in angiogenesis. Binding of VEGFA to its cell-surface receptors FLT1 and VEGFR1 leads to a cascade of signaling pathways: the PI3K–Akt pathway, which leads to increased endothelial cell survival, or the MAPK pathway, which initiates DNA synthesis and cell growth. VEGFA and FLT1 are up-regulated in MCL patients when compared to normal B cells (BtoMCL; Supplementary Table S4).

Eighteen proposed drugs are approved in other types of cancer, and fourteen of them are highlighted by the comparison of MCL cases diagnosed early against advanced (MCL(e)toMCL). Catumaxomab hybrid monoclonal antibody is proposed for the intermediate stage; it binds to antigens CD3 and treats malignant ascites, a condition that develops in cancer patients. Cabazitaxel is an antineoplastic agent used for the treatment of hormone-refractory metastatic prostate cancer [29], which is proposed by the current analysis for the treatment of aggressive MCL (Figure 4). Podofilox, epirubicin and dexrazoxane are DNA topoisomerase 2-alpha (TOP2A) inhibitors and are proposed by the current analysis for treating advanced-stage MCL (BcelltoMCL). Epirubicin and dexrazoxane are cytotoxic anthracyclines. The latter is used to prevent and improve cardiomyopathy and combined with doxorubicin is used for the treatment of metastatic breast cancer [30,31]. Podofilox, found in the podophyllin resin from the roots of podophyllum plants and used for treating genital warts, has potential antineoplastic properties and antiproliferation effects in gastric cancer [32]. Gemcitabine, methotrexate, capecitabine are proposed in our DR analysis for inhibiting the up-regulated thymidylate synthase TYMS, which has a significant role in the de novo mitochondrial biosynthesis of thymidylate, an essential precursor for DNA biosynthesis. Methotrexate, an antifolate used in rheumatoid arthritis (RA) treatment, also has antileukemic effects, and thus it is included in nHL therapeutic regiments [33,34]. Capecitabine is a non-cytotoxic fluoropyrimidine carbamate that is taken orally; it has a synergistic effect when combined with docetaxel in colon cancer [35]; it is also used to treat locally advanced and secondary breast cancer [36]. Gemcitabine is a nucleoside analogue that works by replacing the building blocks of nucleic acids during DNA elongation, leading to cell growth arrest; it has a wide range of antitumor activity as monotherapy or in combination with other anticancer agents administered in NSCLC, ovarian, metastatic breast, and pancreatic cancer [37]. Eribulin, a synthetic analogue of the marine sponge natural product halichondrin B that acts as microtubule inhibitor [38], is selected for targeting tubulin beta-1 chain (TUBB1). Eribulin is used to treat metastatic breast cancer [39] and metastatic or unresectable liposarcoma. Vandetanib is an antineoplastic kinase inhibitor used to treat symptomatic or progressive medullary thyroid cancer in patients with unresectable locally advanced or metastatic disease. Vandetanib is proposed by current DR analysis as an inhibitor of the growth factor VEGFA, which is overexpressed in late MCL stages (III/IV) compared to newly diagnosed MCL cases (MCL(e)toMCL). Another drug proposed for the MCL(e)toMCL transition is regorafenib, an inhibitor of multiple kinases administered in metastatic colorectal cancer patients that have previously received anti-VEGF therapy [40], metastatic gastrointestinal stromal tumor that have been treated with imatinib [41] and hepatocellular carcinoma.

Venetoclax was also in our list of known drug combinations for MCL (Supplementary Table S1) and is used together with ibrutinib [42]. In our prediction of drug combinations of known drugs as a validation of the method (Figure S10), we could not predict effective combinations for venetoclax due to the lack of sufficient connections in the PPIN. Furthermore, venetoclax is included in DrugBank and the BCL2 gene (P10415; Ubiquitin protein ligase binding) is the only listed target of venetoclax, which is known to suppress apoptosis in factor-dependent lymphohematopoietic and neural cells. The BCL2 gene is one of the up-regulated genes listed in (Supplementary Table S4; gene 118) with *p*-values ~ 10^−8^. However, BCL2 was not among the top DEGs, since it was found only in two comparisons: BtoMCL and BtoMCLe. Identifying gene signatures related to apoptosis confirms that our data include relevant markers, strengthening the link between our findings and therapeutic methods.

### 2.6. Exploring Drug Combinations in MCL

We wanted to explore known compound combinations by collecting antitumor regiments and effective multidrug therapies in MCL or other types of lymphoma (Supplementary Table S1B, Supplementary Figure S10B). We ended up with 84 compounds; 44 had more than two protein targets in the human PPIN and were further analyzed. We screened all nine sample comparisons regarding the therapeutics already applied for MCL. Two, eight, five, fifteen, seventeen and twenty out of twenty-eight known drug pairs are predicted to be effective in NtoIS, IStoI, ItoA, BtoMCL(e), BtoMCL and MCL(e)toMCL comparisons, respectively (Supplementary Figure S10B).

A network principal category was assigned to every pair among the 44 known MCL drugs. For BtoMCL and MCLetoMCL sample comparisons, the results are summarized as dual heatmaps in which the compounds known to be effective are highlighted (Supplementary Figure S10B). Vincristine was found to be incompatible with the other agents in the BtoMCL drug screening. These include dexamethasone, etoposide and doxorubicin added to regiments such as R-MACLO-IVAM and R-Hyper-CVAD. On the other hand, in the MCLetoMCL comparison, bortezomib was estimated to be ineffective with vincristine, doxorubicin and dexamethasone, as expected by applied strategies such as VcR-CVAD and VR-CAP cocktails.

**Figure 6:**
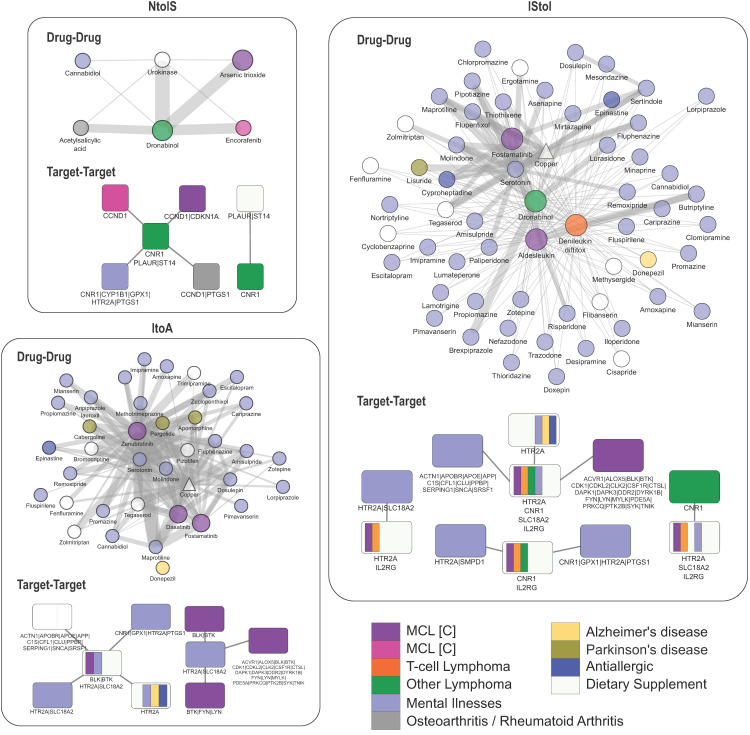
Compound and target combinations in MCL stages. Network-based stratification of drug combinations for the treatment of MCL stages. Networks of effective drug–drug networks and the corresponding target–target combination for the pairwise MCL stage comparisons, NtoIS, IStoI, and ItoA. Drugs are depicted as circles and targets as rectangles. Drugs are colored based on the original disease indication; targets are also colored based on the indications of the respective drugs targeting them (purple: MCL at clinical trials; magenta: MCL; orange: T-cell lymphoma; green: other lymphoma; lilac: psychosis/depressive disorder/bipolar/anxiety/ADHD; gray: osteoarthritis/rheumatoid arthritis; yellow: Alzheimer’s disease; blue: allergic rhinitis; white: dietary supplement).

**Figure 7:**
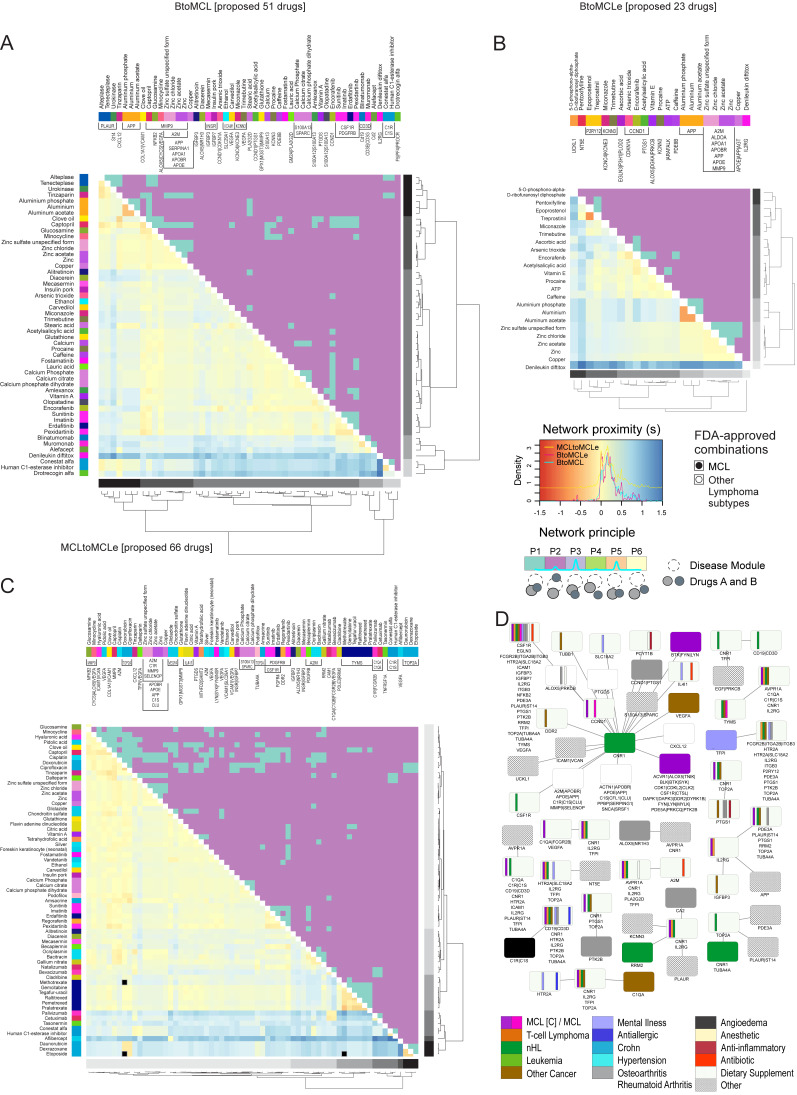
Prediction of effective compound combinations in early and progressed MCL patients. Dual heatmaps present both the network proximity s of drug–drug pairs (lower half) and the network principle of the drug pair given a disease module (upper half). Three drug combination analyses are summarized here, each differing in the selected disease module and the list of proposed drugs assessed: (**A**) BtoMCL (**B**) BtoMCL(e) and (**C**) MCL(e)toMCL. In each analysis, the disease module is defined as the set of top 100 DEGs identified in the respective sample comparison, while only the pairs of the respective proposed drugs are assessed (51, 23, and 66 drugs respectively). Hierarchical clustering of drugs is based on the separation (s) values and clusters are colored in gray shades (rightmost hierarchical tree). Drugs names are colored based on the DEG(s) targets (top color bars) and drug pairs with indications for combined treatment of MCL or other lymphoma subtypes are marked with black or empty circles (see C). (**D**) Target–target network summarizing the effective gene pairs to be treated in early MCL (analysis of BtoMCL(e) transition). The results are based on 190 out of the 388 proposed drugs that can act in combination based on the in-silico screening. Targets are colored based on their original indication(s) and gene names are separated with pipe symbol (|) if they are target of the same drug (magenta: MCL; purple: MCL at clinical trial; dark green: other lymphoma; light green: leukemia; teal: nHL; orange: T-cell lymphoma; light purple: mental illnesses; brown: other cancer; gray: rheumatoid arthritis or osteoarthritis; cyan: Crohn’s disease; red: antibiotic; light yellow: anesthetic; purple: anti-inflammatory; white: dietary supplement).

As a next step, we investigated the pairwise efficacy of the proposed drugs resulting from the DR step at different MCL stages and sample groups. We formed nine disease modules, one per sample comparison (Table 2), each consisting of the respective top 100 DEGs; the NtoIS disease module consisted of 27 genes. We repeated the in-silico drug efficacy screening for every disease module and the respective subset of proposed drugs (Figure 6, Figure 7 and Figure S11). We summarize the proposed dual-drug therapies in three drug–drug networks, consisting of 9, 203 and 120 effective pairs during in situ, intermediate and aggressive MCL stages, respectively (Figure 6). Target–target networks derived from the respective drug combinations were constructed, indicating the proteins/genes to be targeted simultaneously in the potentially successful therapeutic schema. Targeting non-overlapping gene neighborhoods is expected to be potent in treating human diseases. In the current analysis, “cannabinoid receptor 1”, “suppressor of tumorigenicity 14 protein”, and “urokinase plasminogen activator surface receptor” are not in the vicinity of “CCND1” or “cyclin-dependent kinase inhibitor 1”, and this makes them suitable for dual-drug approaches in the in-situ stage. Dronabinol, a synthetic delta-9-THC alleviates nausea caused by cancer chemotherapy, can be combined with encorafenib or arsenic trioxide (Figure 6; NtoIS). In the aggressive stage, inhibition of HTR2A together with the enhancement of tyrosine-protein kinases BTK, BLK, LYN or FYN is expected to be beneficial. In intermediate or aggressive stages, zanubrutinib, dasatinib, fostamatinib, and aldesleukin combined with antipsychotic, antidepressants, or Parkinson’s disease drugs were estimated to have synergistic value.

Finally, we screened the efficacy of 274 out of the 388 compounds, with at least two targets in the PPIN, in patients with early MCL. We constructed a network of effective target pairs, which arose from the respective drug pairs that are in the vicinity of the disease module but targeting different genes (Figure 7D). As suggested by the target–target network, the cannabinoid receptor CNR1 should be targeted alongside genes such as “CCND1”, “cyclin-dependent kinase 1”, “alpha-actinin-1”, “apolipoprotein E”, “apolipoprotein B receptor”, and “tyrosine-protein kinases” BTK, BLK, SYK, FYN, LYN (Figure 7D).

## 3. Discussion

Studying cellular behavior through the prism of gene expression profiling is currently considered a standard procedure, with a wealth of data being deposited in public databases available for reanalysis and meta-analysis. However, exploitation of such data can be challenging due to the different data formats, as well as the heterogeneity of samples and conditions. We analyzed a selected number of datasets using the criterion of the availability of samples taken from healthy individuals, in addition to the well-characterized disease conditions and stages. Large heterogeneous datasets are a prerequisite for the successful investigation of cancer heterogeneity [43]. High-quality Affymetrix datasets of MCL patients at early or progressed stage were included in our pipeline analysis to effectively represent sample diversity in precision medicine. Additional conditions can be analyzed in the future, such as cases harboring mutations other than CCND1 or cases where the disease has not yet developed despite the existence of a chromosomal translocation. Proper sample selection, collection, storage, and preparation is of paramount importance to avoid systematic errors and artificial results that could lead to wrong conclusions and future decisions. Additional bioinformatic tools for data processing, correcting, and integrating should also be employed in the future.

In this study, we described a modular pipeline for the analysis of transcriptomic data that combines different network-based algorithms for exploring differentially expressed genes, enriched pathways, functional modules, and potential drug therapies for MCL at distinct stages. Individual units of the pipeline explore underlying molecular information but from different points of view; the results either led to overlapping conclusions, reinforcing each other, or contributed with uniquely extracted information. Furthermore, by including different implementations of the same type of analyses, we became aware of the limitations of each method. One example is PaintOmics [19], which works with ensemble transcript IDs instead of gene symbols in the case of pathfindR [18] and does not rely on PPIN communities, and thus it accounts for transcripts and not only the protein-coding transcripts.

Taken together, the multifaceted bioinformatic pipeline and the variety of the biological samples (i.e., stepwise morphological stages of MCL or blood samples of patients at distinct stages) brought to light features of a relative abundance of transcripts that relate to the genetic complexity and aggravation of MCL. The potential role of SMAD2/3:SMAD4 and RUNX1 transcriptional regulation and the SUMOylation of transcription cofactors and DNA-related proteins during the first phase of MCL progression (Supplementary Table S5D) is pointed out by two distinct levels of analysis: the pathway enrichment analysis and the co-expression functional module alignment. Progression from early- to late-stage MCL is characterized by SPARC and VEGFA overexpression, identified by the comparative analysis and by the pathway enrichment analysis as members of the “miR-509-3p alteration of YAP1/E” and the “VEGF signaling” pathways, respectively. VEGFA’s role in late-stage MCL is corroborated by the co-expression functional module analysis (Supplementary Figure S8A). Protein-coding genes have undoubtedly the lion’s share in drug target discovery; however, the significance of non-coding transcripts as epigenetic modulators and switches in MCL is evident by our analysis (Supplementary Discussion).

Our approach has identified stage-specific MCL biological pathways, many of which have been previously reported in the literature to be implicated in cancer development and progression [10,11,44,45,46]. The open question is how these pathways are rewired and re-connected in the different cancer cells and cancer types and what the dynamics of this rewiring are. In early MCL, p53, Wnt, Hippo, Hedgehog and prolactin signaling pathways, together with miRNA activity, should be further examined (Supplementary Discussion). In the literature, SPARC overexpression has been correlated with six miRNAs involved in medulloblastoma progression [47], whereas the reciprocal modulation between VEGFA and SPARC proteins has been related to tumor growth [45]. The exact role of these proteins in MCL pathogenesis as well as their use as biomarkers remains to be discovered (Supplementary Discussion).

PE analysis profoundly depends on the established knowledge regarding the molecular circuits in a cell and how this information is recorded in public databases. In rare and not-well-studied diseases, unknown gene networks may be present, and thus it is necessary to employ unbiased computational approaches that explore novel gene relationships characteristic of that disease. Here, we apply functional co-expression module alignment, and we present unique gene networks, characteristic of each MCL stage.

In cancer, the complexity and variability between patients is high, with multiple components participating, and thus combination therapies have higher response rates and can reduce development of drug resistance and relapse [48]. Contemporary MCL therapeutic strategies usually include multiple agents, such as the first-line cytarabine-containing induction chemotherapies (hyper-CVAD and VcR-CVAD regiments; Supplementary Table S1B) [42]. Rarely is monotherapy effective in MCL, such as lenalidomide, an immunomodulator used in R/R MCL [17] (Supplementary Table S1A).

Despite its advantages, multidrug therapy may worsen certain side effects, i.e., toxicity or severe infectious complications (e.g., combinations of BCR kinase inhibitors with chemoimmunotherapy regimens [49]). This underscores the necessity of predicting potentially effective drug combinations and how a drug may interfere with or modulate the action of another drug.

We summarize the potential therapeutic agents per MCL stage, and we propose effective pairwise combinations for further experimental validation (Figure 5 and Figure 6). In cancer, early diagnosis and treatment usually improve the chances of survival and reduce complications, drug resistance and relapse. However, limited treatment options were found for in situ MCL (e.g., encorafenib), whereas more treatment options were proposed for intermediate and aggressive MCL (e.g., aldesleukin, fostamatinib, vincristine, dasatinib).

Antipsychotic drugs, mainly targeting upregulated HTR2A, occupy a large part of the proposed drugs. It has been found that patients treated for schizophrenia have lower incidence of certain types of cancer (respiratory, prostate, bladder cancers [50]) and that serotonin receptor signaling may modulate anti-tumor immunity [51]. As demonstrated, serotonin receptor mRNAs and proteins are expressed across diverse cancer types, and pharmacological inhibition of 5-HT receptors leads to activation of the p53 DNA damage pathway and suppression of MAPK activity [52].

Synergistic enhancement of cancer therapy uses combinations of anti-tumor drugs to reduce toxicity and relapse cases; regiments such as R-hyper-CVAD are standard treatments for MCL (Supplementary Table S1B). We examined efficient drug pairs at different MCL stages by implementing a drug–disease network distance approach [17] and selecting drug pairs assigned to the “complementary exposure” network configuration (Supplementary Figure S10A). We were able to draw conclusions regarding proposed target gene combinations (Figure 6 and Figure 7D), e.g., serotonin or cannabinoid receptors with tyrosine kinases LYN, SYN, FYN, BTK, BLK. These findings supported by our in-silico analysis should be further validated and studied for their clinical impact.

In conclusion, our modular pipeline allowed for the comprehensive exploration of MCL transcriptomic data, which led to a more complete set of potential therapeutic targets. We were able to find individual DEGs in healthy vs. disease conditions, their functional modules and MCL pathogenesis-involved pathways at distinct stages. Moreover, our pipeline examined potential drug therapies, detecting drugs already used for MCL (e.g., lenalidomide) or other types of lymphoma (e.g., pomalidomide, belinostat) as a proof of concept, as well as newly proposed ones. Fostamatinib is proposed for all three MCL stage transitions (Figure 4 and Figure 5) via protein targets, such as SYK, BTK, MAP2K2 or PTK2B, that are affected indirectly. Our work highlights the benefit that can be leveraged by such an approach for identifying high-priority novel, efficacious combination therapies against MCL that should be further validated for their clinical impact.

Despite the advantages of our proposed pipeline, we should emphasize the need for experimental validation of the findings using a combination of approaches (e.g., in vitro and in vivo) and current limitations: (a) prediction of effective drug pairs is limited by the current knowledge of drug–protein and protein–protein interactions that define the structure and density of the PPIN used; (b) gene co-expression networks do not directly imply causal relationships (i.e., cause-and-effect relationships of gene expression); hence, with this method, we are not able to draw conclusions on the origin of the malignancy or design targeted therapies; (c) the proposed pipeline necessitates the preexistence of a batch effect-removal model trained specifically on the platforms used (currently available only for the Affymetrix U133A). From our experience, the removal of batch effect is critical for the correct DEGs to “shine” and removes irrelevant distances produced by tissue type, sex and other aberrations.

## 4. Methods

We applied network-based pathway enrichment algorithms [18,19] and inference methods [53] to independent gene expression data [10,12]. We integrated and reexamined four high-fidelity RNA array experiments available in public databases (ArrayExpress, Gene Expression Omnibus—GEO) including healthy donor samples (Figure 1). The steps of the computation framework were: (1) identification of DEGs, (2) characterization of the significantly affected molecular pathways using pathway enrichment analysis [18,19], (3) construction of gene co-expression networks for the different MCL stages, and 4) exploration of new treatments for MCL stages using available drug repositories (Figure 1).

### 4.1. Selected MCL Gene Expression Studies

We analyzed samples from five GEO dataset series (Figure 1, Supplementary Figure S5): three MCL studies (GSE30189 [10], GSE21452 [20], GSE19243 [22]), one study (GSE45717 [12]) that compares MCL with CCND1 overexpressing asymptomatic monoclonal B-cell lymphocytosis (MALD1) and healthy B cells (GSE65135 [21]) (Table 1, Figure 1, Supplementary Figure S5). Kimura et al. performed cDNA microarray experiments of frozen tissues of lymph node biopsies on Illumina HumanWG-6 v3.0 expression bead chips (GSE30189) [10]. They posit four stepwise morphological grades for MCL, based on the morphological features tissue samples of lymph node biopsies bore: in situ (IS), classical (C), intermediate (I) and aggressive (A) forms. Espinet et al. conducted microarray analyses of five typical MCL cases and of eight healthy individuals using Affymetrix Human Exon 1.0 ST arrays (GSE45717) [12]. Finally, samples from three GEO series (GSE21452, GSE19243, GSE65135) performed on Affymetrix HG U133 Plus2 arrays were analyzed together after we performed blind estimation and correction of batch effects using the BESC algorithm that was described and implemented in R by Varma, S., [54] (see Supplementary Methods). These include RNA profiles of frozen lymph node specimens from 64 MCL patients (GSE21452; GSM536113-76) [20], five early MCL cases (GSE19243; GSM476816-20) [22] and five samples of B cells collected from tonsils of healthy donors (GSE65135; GSM1587845-9) [54].

### 4.2. Support Vector Machine Model Predictions Inform the Grouping of the Samples

Support vector machine (SVM) models were trained using the 500 most variable genes as training features and employing bootstrapping sampling (Supplementary Methods). The original labeling of certain samples was reconsidered based on the SVM model prediction. We trained linear SVM models with a cross-validated ACC = 1 (Supplementary Table S2) by leaving out the classical stage and including the remaining four MCL stages. These models were used to predict the classical stage samples; three out of four samples were systematically predicted as aggressive cases; GSM747378 sample was predicted as aggressive or in situ in 82% and 18% of cases, respectively (Supplementary Table S3). Upon these predictions, we included three classical samples into the aggressive group and completely excluded the GSM747378 sample from the following analyses. Similarly, the GSM536139 sample was incorporated in the newly diagnosed group set of the GSE19243 dataset.

### 4.3. Gene Expression Comparative Analysis

Initially, we sought to examine the similarity between the different samples and sample groups via both multidimensional scaling (MDS) plots, where the average fold change is used as a distance metric, and hierarchical clustering heatmaps of the top variable genes (Supplementary Figures S1–S4; Supplementary Methods). After that, we performed differential gene expression analyses between different sample groups (Table 1, Figure 1, Supplementary Figure S5) using the Limma R package [55] and applying quantile normalization and a *p*-value threshold of 0.05 for the identified DEGs. Every MCL stage of the GSE30189 [10] experiment was compared to its preceding stage (NtoIS: normal to in situ; IStoI: classical to intermediate; ItoA: intermediate to aggressive; Table 1, Supplementary Figure S5), whereas in the GSE45717 [12] dataset, the five typical MCL samples were compared to the eight healthy ones (Figure 1; HtoMCLx). Finally, we compared the 64 MCL samples (GSE21452) and the early MCL cases (GSE19243) against five B-cell extracts from tonsils of healthy people (GSE65135)(BtoMCL, BtoMCL(e), MCL(e)toMCL; Table 1, Supplementary Figure S5).

### 4.4. Pathway Enrichment Analysis

We analyzed the DEGs from the perspective of biological pathways. PathfindR [18] and PaintOmics [19] bioinformatic tools were employed to perform pathway enrichment analysis (Figure 1, Supplementary Figure S5). Both pathfindR and PaintOmics use the *p*-values of the comparative analysis step to calculate biological pathway enrichment values. PaintOmics accepts only ensembl identifiers, and therefore preprocessing of these identifiers was necessary for several transcripts that lacked such an identifier. The output of PaintOmics consists of several types of graphs (e.g., pie chart of gene ontology annotations), visualizations (e.g., enriched pathways where genes are colored based differential expression level) and functionalities available via a web interface. Enrichment of a pathway is evaluated by a *p*-value. PathfindR’s input is a table where every detected transcript is represented by its symbol (gene name) and accompanied by a logarithmic value of the fold change and a respective *p*-value of the comparative analysis. Via an inner process, pathfindR first maps symbol names to synonyms. Next, active subnetworks (i.e., clusters of genes with similar *p*-values) are detected using three main algorithms and several random initiations of these searches. Pathway enrichment is examined in the context of these active subnetworks. In contrast to PaintOmics, pathfindR provides three different values that evaluate the significance of enrichment: the *p*-values, the *FE*, and the number of occurrences (number of searches where a pathway was found to be enriched). Selected enriched pathways identified in the different sample group comparisons were juxtaposed via a hierarchical clustering heatmap of pathfindR’s *FE* output values (Supplementary Table S5C, Figure 2B). Pathway–pathway networks were also constructed for the same selected pathways (Supplementary Figure S6), where edges connected pathways with common genes. Genes and biological states were also clustered using hierarchical clustering. Except for the in-situ stage, the three final stages were also compared with the normal state (NtoIS, NtoI, NtoA; Table 1).

### 4.5. Co-Expression Network Construction

We applied mutual information network inference methods to construct co-expression networks of different sample groups. The MRNETB (Maximum Relevance Minimum Redundancy Backward) network inference algorithm of the minet R/Bioconductor package [56] was employed, where the mutual information is an estimation based on *k* nearest neighbors, as proposed by Krakov et al. [57] and implemented in the parmigene R package. The co-expression networks of the sample groups were constructed, starting with the batch effect corrected and quantile-normalized transcriptional profiles. First, the mutual information between every pair of transcripts was calculated using entropy estimates from K-nearest-neighbor distances [57]. The estimated mutual information values were not normalized via Fisher’s *z* transformation as described in Zhang et al.; they were used as an input for the MRNETB algorithm to infer the networks using the maximum relevance/minimum redundancy criterion [56]. Hard thresholding was applied to transform the weighted networks to unweighted by taking a quantile as a threshold, below which the values were set to zero. The quantile thresholds were different for each co-expression network, ranging from 93% to 98%. Therefore, co-expression networks were represented by their adjacency matrices $A_{k}$ where $A_{k}\left(i,j\right)=1$ when there is an edge between genes *i* and *j*. Prior to module identification, the vertices, which were unconnected in both networks, were removed.

### 4.6. Identifying Functional Modules in Co-Expression Networks

The co-expression networks were aligned in terms of functional modules using the spectral clustering approach of Zhang et al. [24]. Spectral clustering is a graph-based, unsupervised learning method that incorporates other known clustering algorithms, usually *k*-means. It performs with the same time module identification and alignment between different networks (here co-expression networks). Transcription values remain unlabeled for the clustering algorithm relative to the co-expression network they originate from and are grouped to a given number of clusters (*k*). This spectral clustering approach integrates adjacency matrices of more than two networks, and hence it can compare more than two networks. Each gene is represented as a node as many times as the number of networks compared (here, we focused only on pairs of co-expression networks). A cluster in the first network and its counterpart cluster in the second network may include different genes, but the final modules are constructed by the union of those genes. The number of modules, i.e., the number of clusters, is decided based on the eigenvalue gap (Supplementary Figure S7). We confined the size of the aligned modules from 8 to 800 nodes (Supplementary Figure S5), and we excluded the unconnected subnetworks from the final modules. As a means of representing the changes between the two networks, we used the fold of change of the co-expression value that was assigned to the edges (Figure 3, Supplementary Figure S8). Co-expression modules were visualized and merged using Cytoscape software (v3.9.1) [58]. Finally, we sought to find the enriched terms in the identified modules, and so we performed GSEA analyses using the clusterProfiler R package. We searched for enriched Wiki and KEGG pathways, but also Gene Ontology terms (biological process and molecular function) (Supplementary Table S7A).

### 4.7. Drug Repurposing

We explored new potential treatments for each MCL stage using the drug–target information stored in DrugBank database [26]. Totally, 15,163 drug–gene interactions of approved drugs were available, of which 8680 were of type “target” (DrugBank organizes drug–gene interactions into four main types: target, carrier, transporter, and enzyme). In 3199 of the drug–target interactions, the action of the drug upon the target was characterized (e.g., inhibitor, partial agonist, substrate, binder). The drug actions are accessible through the full xml DrugBank database (32 types). To define the proposed drugs, we first selected the top 100 DEGs of each sample group comparison (Supplementary Figure S5). Then, drug–target edges were filtered out in case the type of action was the opposite of the preferred one, as dictated by the fold-change direction (Supplementary Figure S5f; Supplementary Methods). In cases of significantly up-regulated genes, targeting drugs that enhance their activity (i.e., activators, agonists, partial agonists) were excluded from the list. Contrarily, if a gene was down-regulated, then “inhibitors” and “antagonists” were not considered as potential treatments.

### 4.8. Assessing Drug Combination Efficacy

Combination therapies has been proven more effective than monotherapy regimens. They allow lower doses and reduce the risk of toxicities or adverse effect in patients [42,59]. We sought to explore synergistic drug pairs in MCL by implementing a recently proposed network-based stratification method [17] that was tested on antihypertensive drug combinations. This method is based on network proximity metrics that reflect the topological overlap between drug modules and disease modules within the human protein–protein interaction network (PPIN).

The in-silico screening of drug-pair efficacy described by Cheng et al. defines two network proximity measures, the separation (s) and the z-score, to estimate the closeness of drugs and of drug and disease modules, respectively. They define the disease module as the set of genes that are related to a certain disease. These genes are usually found in proximity to the human PPIN. Both separation (s) and the z-score (z) distance measures rely on the calculation of shortest paths between pairs of proteins within the human PPIN (Supplementary Methods). The value of the separation (s) measure reflects the proximity of two drugs: if negative, the two drugs have overlapping targets; otherwise, they have distinct targets. In the same manner, when the value of the z-score is negative, then the drug targets proteins of the disease module. After calculating the separation values between all pairs of drugs of interest and the respective z-scores between drugs and the disease module, the method decides for each pair one of the six network principles based on the network proximity between the drug’s targets and the disease module (Supplementary Figure S10A). The network principle is decided based on three values: the separation ($s_{AB}$) of drugs A and B and the z-scores $z_{A}$ and $z_{B}$ between drugs A, B, and the disease module (Supplementary Figure S10A). As concluded by Cheng et al. [17], the most effective drug combinations fall under the second principle, the “complementary exposure”, where both drugs overlap with the disease module, but not with each other (Figure S10A).

For the analysis, we used the human PPIN network provided by Cheng et al. [17], and we employed an in-house implementation of the Dijkstra algorithm to calculate the shortest paths between nodes in the network. We defined four disease modules, each consisting of the top 200 DEGs of the between-stage comparisons (NtoIS, IStoI, ItoA). Of the 388 proposed drugs, we discarded those with fewer than two targets present in the human PPIN for reasons of statistical validity (275 remained). Multidrug therapeutic strategies currently used in the treatment of MCL were collected from the Drug Combination Database (DCDB) [60] and from the literature (Supplementary Table S1B).

## Figures and Tables

**Table 1:** Datasets used in gene co-expression network analysis.

Data Series	Samples	Publication	Platform	Type of Samples	Stages	No of Samples	Control	Comparisons
**E-GEOD-45717**	GSM1112430-34 GSM1112435-40,42-43	(Espinet et al., 2014) [14]	Affymetrix Exon 1.0 ST Array	Blood	No	▪8 healthy ▪5 MCL patients	PB B cells	HtoMCLx
**E-GEOD-30189**	GSM747367-87	(Kimura et al., 2013) [10]	Illumina HumanWG-6 v3.0 expression bead chip	Lymph node tissue	Yes	4 normal 4 in situ 4 classical 4 intermediate 5 aggressive	Normal B cells	NtoIS IStoI ItoA NtoI NtoA
**E-GEOD-21452**	GSM536113-76	(Hartmann et al., 2010) [20]	Affymetrix Human Genome U133 Plus 2.0 Array	Lymph node tissue	No	▪64 MCL patients		BtoMCL
**E-GEOD-65135**	GSM1587845-54	(Newman et al., 2015) [21]		Tonsils		▪5 healthy	Healthy B cells from tonsils	
**E-GEOD-19243**	GSM476816-20	(Leshchenko et al., 2010) [22]		CD19+ fractions from peripheral blood		▪5 MCL newly diagnosed patients		BtoMCL(e) MCL(e)toMCL

**Table 2:** Number of enriched pathways per MCL stage transition and MCL vs. healthy white blood cells.

Comparison		Bioinformatic Tool				
		pathfindR (R Package)				PaintOmics (web)
		#Enriched Terms				
	DEGs	KEGG	Reactome	BioCarta	GO (all)	KEGG
*NtoIS*	27	24	7	5	3	2
*IStoI*	1176	138	433	82	187	19
*ItoA*	2677	158	663	104	462	11
*NtoI*	1659	150	556	108	310	39
*NtoA*	2913	169	731	123	534	21
*HtoMCLx*	5467	202	870	166	759	45
*BtoMCL(e)*	6588	202	851	162	729	42
*BtoMCL*	9510	194	814	152	747	7
*MCL(e)toMCL*	9083	195	821	160	713	12

## Data Availability

The data and R code used for the calculation of network proximities of drug–disease and drug–drug pairs are available at github in the following link https://github.com/gorfoula/cocktails (accessed on 8 February 2023). In-house implementation of common co-expression module identification is available at github in the following link https://github.com/gorfoula/CoexModules (accessed on 6 March 2023).

## Supplementary Materials

The following supporting information can be downloaded at https://www.mdpi.com/article/10.3390/ijms25137298/s1.

## References

[1] Alaggio R., Amador C., Anagnostopoulos I., Attygalle A.D., Araujo I.B.d.O., Berti E., Bhagat G., Borges A.M., Boyer D., Calaminici M. (2022). The 5th edition of the World Health Organization Classification of Haematolymphoid Tumours: Lymphoid Neoplasms. Leukemia.

[2] Campo E., Jaffe E.S., Cook J.R., Quintanilla-Martinez L., Swerdlow S.H., Anderson K.C., Brousset P., Cerroni L., de Leval L., Dirnhofer S. (2022). The International Consensus Classification of Mature Lymphoid Neoplasms: A report from the Clinical Advisory Committee. Blood.

[3] Navarro A., Beà S., Jares P., Campo E. (2020). Molecular Pathogenesis of Mantle Cell Lymphoma. Hematol. Oncol. Clin. North Am..

[4] Salaverria I., Royo C., Carvajal-Cuenca A., Clot G., Navarro A., Valera A., Song J.Y., Woroniecka R., Rymkiewicz G., Klapper W. (2013). CCND2 rearrangements are the most frequent genetic events in cyclin D1(-) mantle cell lymphoma. Blood.

[5] Albero R., Enjuanes A., Demajo S., Castellano G., Pinyol M., Garcia N., Capdevila C., Clot G., Suarez-Cisneros H., Shimada M. (2018). Cyclin D1 overexpression induces global transcriptional downregulation in lymphoid neoplasms. J. Clin. Investig..

[6] Vogt N., Dai B., Erdmann T., Berdel W.E., Lenz G. (2017). The molecular pathogenesis of mantle cell lymphoma. Leuk Lymphoma.

[7] Yi S., Yan Y., Jin M., Bhattacharya S., Wang Y., Wu Y., Yang L., Gine E., Clot G., Chen L. (2022). Genomic and transcriptomic profiling reveals distinct molecular subsets associated with outcomes in mantle cell lymphoma. J. Clin. Investig..

[8] Jain P., Wang M. (2019). Mantle cell lymphoma: 2019 update on the diagnosis, pathogenesis, prognostication, and management. Am. J. Hematol..

[9] Silkenstedt E., Dreyling M. (2023). Mantle cell lymphoma-Update on molecular biology, prognostication and treatment approaches. Hematol. Oncol..

[10] Kimura Y., Arakawa F., Kiyasu J., Miyoshi H., Yoshida M., Ichikawa A., Niino D., Sugita Y., Okamura T., Doi A. (2013). The Wnt signaling pathway and mitotic regulators in the initiation and evolution of mantle cell lymphoma: Gene expression analysis. Int. J. Oncol..

[11] Merolle M.I., Ahmed M., Nomie K., Wang M.L. (2018). The B cell receptor signaling pathway in mantle cell lymphoma. Oncotarget.

[12] Espinet B., Ferrer A., Bellosillo B., Nonell L., Salar A., Fernandez-Rodriguez C., Puigdecanet E., Gimeno J., Garcia-Garcia M., Vela M.C. (2014). Distinction between asymptomatic monoclonal B-cell lymphocytosis with cyclin D1 overexpression and mantle cell lymphoma: From molecular profiling to flow cytometry. Clin. Cancer Res..

[13] Lin C.Y., Loven J., Rahl P.B., Paranal R.M., Burge C.B., Bradner J.E., Lee T.I., Young R.A. (2012). Transcriptional amplification in tumor cells with elevated c-Myc. Cell.

[14] Hartmann E., Fernandez V., Moreno V., Valls J., Hernandez L., Bosch F., Abrisqueta P., Klapper W., Dreyling M., Hoster E. (2008). Five-gene model to predict survival in mantle-cell lymphoma using frozen or formalin-fixed, paraffin-embedded tissue. J. Clin. Oncol..

[15] Fernandez V., Salamero O., Espinet B., Sole F., Royo C., Navarro A., Camacho F., Bea S., Hartmann E., Amador V. (2010). Genomic and gene expression profiling defines indolent forms of mantle cell lymphoma. Cancer Res..

[16] Jadamba E., Shin M. (2016). A Systematic Framework for Drug Repositioning from Integrated Omics and Drug Phenotype Profiles Using Pathway-Drug Network. Biomed Res. Int..

[17] Cheng F., Kovács I.A., Barabási A.-L. (2019). Network-based prediction of drug combinations. Nat. Commun..

[18] Ulgen E., Ozisik O., Sezerman O.U. (2018). pathfindR: An R Package for Pathway Enrichment Analysis Utilizing Active Subnetworks. bioRxiv.

[19] Garcia-Alcalde F., Garcia-Lopez F., Dopazo J., Conesa A. (2011). Paintomics: A web based tool for the joint visualization of transcriptomics and metabolomics data. Bioinformatics.

[20] Hartmann E.M., Campo E., Wright G., Lenz G., Salaverria I., Jares P., Xiao W., Braziel R.M., Rimsza L.M., Chan W.C. (2010). Pathway discovery in mantle cell lymphoma by integrated analysis of high-resolution gene expression and copy number profiling. Blood.

[21] Newman A.M., Liu C.L., Green M.R., Gentles A.J., Feng W., Xu Y., Hoang C.D., Diehn M., Alizadeh A.A. (2015). Robust enumeration of cell subsets from tissue expression profiles. Nat. Methods.

[22] Leshchenko V.V., Kuo P.Y., Shaknovich R., Yang D.T., Gellen T., Petrich A., Yu Y., Remache Y., Weniger M.A., Rafiq S. (2010). Genomewide DNA methylation analysis reveals novel targets for drug development in mantle cell lymphoma. Blood.

[23] Zhang S. (2018). Comparisons of gene coexpression network modules in breast cancer and ovarian cancer. BMC Syst Biol.

[24] Zhang S., Zhao H., Ng M.K. (2015). Functional Module Analysis for Gene Coexpression Networks with Network Integration. IEEE/ACM Trans. Comput. Biol. Bioinform..

[25] Martens M., Ammar A., Riutta A., Waagmeester A., Slenter D.N., Hanspers K., Miller R.A., Digles D., Lopes E.N., Ehrhart F. (2021). WikiPathways: Connecting communities. Nucleic Acids Res..

[26] Wishart D.S., Feunang Y.D., Guo A.C., Lo E.J., Marcu A., Grant J.R., Sajed T., Johnson D., Li C., Sayeeda Z. (2018). DrugBank 5.0: A major update to the DrugBank database for 2018. Nucleic Acids Res..

[27] Friedberg J.W., Sharman J., Sweetenham J., Johnston P.B., Vose J.M., Lacasce A., Schaefer-Cutillo J., De Vos S., Sinha R., Leonard J.P. (2010). Inhibition of Syk with fostamatinib disodium has significant clinical activity in non-Hodgkin lymphoma and chronic lymphocytic leukemia. Blood.

[28] Younes A., Ansell S., Fowler N., Wilson W., de Vos S., Seymour J., Advani R., Forero A., Morschhauser F., Kersten M.J. (2017). The landscape of new drugs in lymphoma. Nat. Rev. Clin. Oncol..

[29] Tsao C.-K., Cutting E., Martin J., Oh W.K. (2014). The role of cabazitaxel in the treatment of metastatic castration-resistant prostate cancer. Ther. Adv. Urol..

[30] Seymour L., Bramwell V., Moran L.A. (1999). Use of dexrazoxane as a cardioprotectant in patients receiving doxorubicin or epirubicin chemotherapy for the treatment of cancer. The Provincial Systemic Treatment Disease Site Group. Cancer Prev. Control.

[31] Langer S.W. (2014). Dexrazoxane for the treatment of chemotherapy-related side effects. Cancer Manag. Res..

[32] An J., Liu Y., Duo S., Ma X., An L., Yan Y., Ji D., Yan Y., Cheng Q., Su Z. (2021). Podofilox suppresses gastric cancer cell proliferation by regulating cell cycle arrest and the c-Myc/ATG10 axis. Exp. Ther. Med..

[33] Fleming M., Huang Y., Dotson E., Bond D.A., Reneau J., Epperla N., Alinari L., Brammer J., Christian B.A., Baiocchi R.A. (2022). Feasibility of high-dose methotrexate administered on day 1 of (R)CHOP in aggressive non-Hodgkin lymphomas. Blood Adv..

[34] Gomez G.A., Stutzman L., Moayeri H., Shimaoka K., Plager J., Han T., Naeher C., Henderson E. (1982). Combinations of methotrexate (COP or CHOP) in the treatment of previously untreated and treated lymphomas. Cancer Treat. Rep..

[35] Pronk L.C., Vasey P., Sparreboom A., Reigner B., Planting A.S., Gordon R.J., Osterwalder B., Verweij J., Twelves C. (2000). A phase I and pharmacokinetic study of the combination of capecitabine and docetaxel in patients with advanced solid tumours. Br. J. Cancer.

[36] Iizumi S., Shimomura A., Shimoi T., Sudo K., Noguchi E., Yonemori K., Shimizu C., Fujiwara Y., Tamura K. (2018). Efficacy of capecitabine in patients with locally advanced or metastatic breast cancer with or without prior treatment with fluoropyrimidine: A retrospective study. Cancer Chemother. Pharmacol..

[37] Barton-Burke M. (1999). Gemcitabine: A pharmacologic and clinical overview. Cancer Nurs..

[38] Smith J.A., Wilson L., Azarenko O., Zhu X., Lewis B.M., Littlefield B.A., Jordan M.A. (2010). Eribulin binds at microtubule ends to a single site on tubulin to suppress dynamic instability. Biochemistry.

[39] O’Shaughnessy J., Cortes J., Twelves C., Goldstein L.J., Alexis K., Xie R., Barrios C., Ueno T. (2020). Efficacy of eribulin for metastatic breast cancer based on localization of specific secondary metastases: A post hoc analysis. Sci. Rep..

[40] Aljubran A., Elshenawy M.A., Kandil M., Zahir M.N., Shaheen A., Gad A., Alshaer O., Alzahrani A., Eldali A., Bazarbashi S. (2019). Efficacy of Regorafenib in Metastatic Colorectal Cancer: A Multi-institutional Retrospective Study. Clin. Med. Insights. Oncol..

[41] Kelly C.M., Gutierrez Sainz L., Chi P. (2021). The management of metastatic GIST: Current standard and investigational therapeutics. J. Hematol. Oncol..

[42] Schieber M., Gordon L.I., Karmali R. (2018). Current overview and treatment of mantle cell lymphoma. F1000Res.

[43] Dagogo-Jack I., Shaw A.T. (2018). Tumour heterogeneity and resistance to cancer therapies. Nat. Rev. Clin. Oncol..

[44] Chang Y., Fu X.-R., Cui M., Li W.-M., Zhang L., Li X., Li L., Sun Z.-C., Zhang X.-D., Li Z.-M. (2019). Activated hippo signal pathway inhibits cell proliferation and promotes apoptosis in NK/T cell lymphoma cells. Cancer Med..

[45] Podhajcer O.L., Benedetti L., Girotti M.R., Prada F., Salvatierra E., Llera A.S. (2008). The role of the matricellular protein SPARC in the dynamic interaction between the tumor and the host. Cancer Metastasis Rev..

[46] Xiong W., Yi S., Yan Y., Li Z., Liu W., Lv R., Yu Z., Zou D., Qiu L. (2019). Inhibiting the Hippo Signaling Pathway Key Molecule YAP Suppresses Mantle Cell Lymphoma Proliferation By Regulating Multiple Pathogenrelated Signaling Pathways. Blood.

[47] Ahir B.K., Elias N.M., Lakka S.S. (2017). SPARC overexpression alters microRNA expression profiles involved in tumor progression. Genes Cancer.

[48] He B., Lu C., Zheng G., He X., Wang M., Chen G., Zhang G., Lu A. (2016). Combination therapeutics in complex diseases. J. Cell. Mol. Med..

[49] Rodgers T.D., Barr P.M. (2018). Pitfalls of Combining Novel Agents in Lymphoma. Curr. Treat. Options Oncol..

[50] Shaw V., Srivastava S., Srivastava S.K. (2021). Repurposing antipsychotics of the diphenylbutylpiperidine class for cancer therapy. Semin. Cancer Biol..

[51] Karmakar S., Lal G. (2021). Role of serotonin receptor signaling in cancer cells and anti-tumor immunity. Theranostics.

[52] Ballou Y., Rivas A., Belmont A., Patel L., Amaya C.N., Lipson S., Khayou T., Dickerson E.B., Nahleh Z., Bryan B.A. (2018). 5-HT serotonin receptors modulate mitogenic signaling and impact tumor cell viability. Mol. Clin. Oncol..

[53] Bourdakou M.M., Athanasiadis E.I., Spyrou G.M. (2016). Discovering gene re-ranking efficiency and conserved gene-gene relationships derived from gene co-expression network analysis on breast cancer data. Sci. Rep..

[54] Varma S. (2020). Blind estimation and correction of microarray batch effect. PLoS ONE.

[55] Ritchie M.E., Phipson B., Wu D., Hu Y., Law C.W., Shi W., Smyth G.K. (2015). limma powers differential expression analyses for RNA-sequencing and microarray studies. Nucleic Acids Res..

[56] Meyer P.E., Lafitte F., Bontempi G. (2008). minet: A R/Bioconductor Package for Inferring Large Transcriptional Networks Using Mutual Information. BMC Bioinform..

[57] Kraskov A., Stögbauer H., Grassberger P. (2004). Estimating mutual information. Phys. Rev. E.

[58] Shannon P., Markiel A., Ozier O., Baliga N.S., Wang J.T., Ramage D., Amin N., Schwikowski B., Ideker T. (2003). Cytoscape: A software environment for integrated models of biomolecular interaction networks. Genome Res..

[59] DeVita V.T.J., Young R.C., Canellos G.P. (1975). Combination versus single agent chemotherapy: A review of the basis for selection of drug treatment of cancer. Cancer.

[60] Liu Y., Wei Q., Yu G., Gai W., Li Y., Chen X. (2014). DCDB 2.0: A major update of the drug combination database. Database.

