# Supplementary material for: Insight into Mantle Cell Lymphoma Pathobiology, Diagnosis, and Treatment Using Network-Based and Drug-Repurposing Approaches
Source: Int J Mol Sci. 2024 Jul 2;25(13):7298. doi: 10.3390/ijms25137298 (PMC11242097; doi:10.3390/ijms25137298)
Supplement: Supplementary file 1 [file ijms-25-07298-s001.zip › ijms-2969548-supplementary.pdf]

Supplementary Information

Table of contents

Supplementary Figures

Supplementary Figure S1: MDS analysis of the MCL stages (GSE30189) dataset 10
Supplementary Figure S2: MDS analysis of the MCL tissues (GSE45717) dataset..11
Supplementary Figure S3: Affymetrix Human Genome U133 Plus 2.0 array data 12
Supplementary Figure S4: Hierarchical clustering heatmaps of transcription profiles...13
Supplementary Figure S5 Analytic pipeline of MCL gene expression data analysis 14
Supplementary Figure S6: Pathway-pathway networks at different MCL stages...16
Supplementary Figure S7: Spectral clustering analysis – Eigenvalue Curves 17
Supplementary Figure S8: Co-expression network and module alignment: Affymetrix U133 Plus 2.0 array data.....18
Supplementary Figure S9: Summarizing proposed MCL drug's features 19
Supplementary Figure S10: Validation of compound pair prediction using known MCL regiments..........................20
Supplementary Figure S11: Predicted drug combinations at different MCL stages. 21

Supplementary Tables

Supplementary Table S1: Short overview of existing therapies, repurposed drugs and deregulated biological pathways in MCL 23
Supplementary Table S2: SVM models trained for the separation of MCL stages. 23
Supplementary Table S3: Predicting Classical Stage samples using linear SVM classifier. 23
Supplementary Table S4: Catalogue of the top differentially expressed transcripts. 23
Supplementary Table S5: Enriched pathways in MCL...23
Supplementary Table S6 Proposed drug for MCL treatment..23
Supplementary Table S7 Aligned common modules discovered with spectral clustering. 23

Supplementary Materials and Methods

Batch Effect Removal: We merged results of 74 samples taken from three independent experiments (E-GEOD-21452, E-GEOD-65135 and E-GEOD-19243) that were performed on the same microarray platform (Affymetrix Human Genome U133 Plus 2.0 [HG-U133_Plus_2]; see **Table 1**). To do so, we employed the Batch Effect Signature Correction (BESC) algorithm of Varma S[1] implemented by the besc package in R. The BESC method corrects the systematic biases between microarrays datasets generated by different labs at different times or under different experimental conditions. In this approach it is assumed that common effect of experimental conditions is shared between datasets. The Batch Effect Signatures (BES), which are an orthogonal set of vectors, that were learned on a carefully selected reference set of Affymetrix U133 Plus2 datasets. Based on the learned batch effect signatures and given a new dataset the technical variation (batch effect) is removed, and the measurements are corrected blindly.

Sample Classification: We trained SVM models to distinguish (a) MCL newly diagnosed samples (MCL(e); E-GEOD-19243) from MCL- mixed stages (E-GEOD-21452) and (b) MCL stages Normal, In Situ, Intermediate, Aggressive stages (E-GEOD-30189). We employed the kernlab R package for SVM model training; tested degree-d polynomial (d=2, 3, 4) and radial basis SVM kernels (Table S2). SVM models were trained using the top-500 most variable genes as training features. Bootstrapping sampling method was applied (number of iterations N = 100), where 25% of each stage sample group (N, IS, I, A) and 20 out of 64 MCL samples were randomly left out (in each iteration), as test sets in (a) and (b) correspondingly. In case (a), we used the trained SVM models to predict the class of the GSM536139 sample (E-GEOD-21452), which exhibited similar profile with five newly diagnosed patients of the E-GEOD-19243 dataset (GSM476816-20; Figure S3B); in all trained models the GSM536139 is classified to the MCL newly diagnosed group. In case (b), we left out the Classical stage samples (GSM747375-78) from training and we predicted their class using the best performing classifier, the linear SVM model (Table S3).

Spectral Clustering: In its simplest version, spectral clustering calculates the Laplacian matrix L of an adjacency matrix A and uses the eigenvectors of the M smallest eigenvalues as data points to be clustered (usually with k-means). In Zhang's approximation [2] a new matrix C is created with the Laplacian (L_k) matrices of all the networks to be integrated, at its diagonal. In that way multiple co-expression networks that correspond to different biological conditions are integrated. Each gene is represented by two or more experimental values, depending on how many networks (biological states) we choose to align.

$C = \begin{bmatrix} L_i & \mathbf{0} \\ \mathbf{0} & L_j \end{bmatrix} - \beta \begin{bmatrix} \mathbf{0} & I_n \\ I_n & \mathbf{0} \end{bmatrix}$, where I_n is the identity matrix, $L_k = D_k - A_k$, A_k is the adjacency matrix, and D_k is a diagonal matrix of the node degrees of the co-expression network k

The parameter β controls the balance between inter- and intra- network connections of the vertices. The larger the value of β, the more connected the two co-expression networks tend to be, and their vertices tend to belong to the same cluster. In the $\beta = 0$ case, the algorithm finds the individual modules of each co-expression network and not the common ones. When $\beta > 0$, vertices that are highly connected in one of the two networks, tend to cluster together with their counterparts in the other network, even if they are loosely connected in that network. Prior to module detection we also examined the node degree distributions at various quantile thresholds; our network pairs turned out to have almost equal average node degrees. Hence, we assumed that the connectivity was comparable among each network pair, and we were able to directly set the connection weight, $\beta = 1$, as suggested by Zhang et al.

The number of clusters M was decided based on the eigenvalue curve and was selected as the number k that maximizes the eigengap (difference between consecutive eigenvalues). The larger this eigengap is, the closer the eigenvectors of the ideal case and spectral clustering is closer to optimal. M does not correspond to the number of clusters in either of the two networks, but rather to the union of clusters in both networks, because when $\beta > 0$, the unconnected vertices in one network tend to be clustered together depending on the other network. As a result, a cluster of the first network, and its counterpart cluster in the second network, may include different genes. The final module comprises of the union of those genes and the unconnected sub-networks are removed.

Next, spectral clustering uses the first M eigenvectors as clustering parameters, and any clustering algorithm can be applied. We tested k-means and complete hierarchical clustering with Euclidean distance as closeness metric. The number of clusters in the k-means clustering were decided based on the number of eigenvalues of the matrix C that were near zero (**Figure S6, TableS7**). K-means is known to be sensitive to the initialization step, where data points are randomly assigned to clusters. We performed 100 iterations of k-means clustering. and we calculated the average number of aligned modules. As aligned modules, we defined those communities containing from eight to 800 DEGs nodes, whereas nodes not connected to the main module were discarded (**Figure 3, S8**). The aligned modules of all

iterations were merged and compared in terms of common nodes. As it turned out k-means spectral clustering was not repeatable at high k values leading to largely disjoint modules between iterations. Therefore, we decided that hierarchical clustering was more appropriate in network comparisons, where the estimated number of modules was larger than 50.

Drug-gene networks: The top 100 DEGs with p-value less than 5% of each comparison performed (NtoIS, IStoI, ItoA, NtoI, NtoA, HtoMCLx, BtoMCL, BtoMCL(e), MCLtoMCL(e)), were used to retrieve potential drug treatments targeting the specific MCL or control sample groups. We constructed the comprehensive drug-gene network using the information retrieved from DrugBank [3], regarding the FDA approved drugs and their gene targets. Sub-selections of drug-gene subnetworks for the various sample group comparisons (NtoIS, MCLtoMCL(e), etc.), were visualized using the Cytoscape platform [4].

Pathway-gene network: We constructed the comprehensive pathway-gene bipartite graph G (V_1, V_2), where the vertices V_1 and V_2 are the set of KEGG pathways and their gene members correspondingly [5]. Genes-pathway relations were retrieved from KEGG using the KEGGREST R package.

Pathway-Pathway network: We constructed the complete pathway-pathway graph G (V, E, W), where V is the set of KEGG pathways, E is the set of edges connecting pathways that share common genes, and W the edge weights equal to the number of common genes. The pathway-gene pairs were retrieved using KEGGREST R package. Using in-house R scripts, we counted the common genes between every pair of pathways and constructed a 330x330 adjacency matrix. Pathway pairs lacking common genes were excluded (no edge between the respective vertices).

Filtering of proposed drugs based on drug action and direction of regulation: Drug-gene (Figure 4) relations were reconsidered given the documented action of a drug on its target and the type of regulation (up or down) observed experimentally for the specific target. Based on the following five rules, drug-gene pairs were excluded from the final proposed catalogue:

**Action**	**Regulation (logFC sign)**
agonist	up
Partial agonist	up
antagonist	down
inhibitor	down
inducer	up

Multidimensional scaling (MDS): The MDS analysis was performed using the plotMDS function of the limma R package, where the average fold change was used as a distance metric. Expression values were first transformed to cpm values (counts per million) using the edgeR R package. The top 200 or 500 variable genes were used for the analysis. Distances on the plot can be interpreted as leading log2-fold-change, meaning the typical (root-mean-square) log2-fold-change, between the samples for the genes that distinguish those samples (**Figure S1-S3B**).

Network proximity of drugs-drug pairs: Network proximity between two drugs d_A and d_B, as reflected in the localization of target genes in the human PPIN, was calculated using the separation measure introduced by Cheng et al. [6],

$$s_{AB} = d\langle A, B\rangle - \frac{d\langle A, A\rangle + d\langle B, B\rangle}{2}$$

where A and B are the sets of target genes of drugs d_A and d_B correspondingly. The separation measure compares the mean shortest distance within the PPIN, between the targets of each drug, $d\langle A, A\rangle$ and $d\langle B, B\rangle$, to the mean shortest distance $d\langle A, B\rangle$, between A–B target pairs.

Network proximity of drugs-disease pairs: The distance between drug targets and disease genes in the PPIN was calculated as described in Cheng et al. [6]. Let X be the set of drug targets of drug d_X, Y the set of genes related to the disease (or disease module), and d(x,y) is the shortest path length between nodes x and y in the human protein-protein interaction network (PPIN), then the network proximity of the disease module Y and the drug d_X is defined as:

$$d(X, Y) = \frac{1}{\|X\|} \sum_{x \in X} min_{y \in Y} d(x, y)$$

The significance of the $d(X,Y)$ measure is evaluated comparing it to the reference distance distribution, corresponding to the expected network topological distance between two randomly selected groups of proteins, matched to size and degree (connectivity) distribution, as the original disease genes and drug targets in the human PPIN. Here, the procedure was repeated 100 times. The mean distance ($\tilde{d}$) and standard deviation (σ_d) of the reference distribution were used to calculate a z-score (z):

$$z = \frac{d - \tilde{d}}{\sigma_d}$$

Shortest paths between nodes: We implemented Dijkstra algorithm in C and R code to estimate the shortest paths from a starting to a destination node. The human PPIN is an undirected graph where all weights were considered as equal to "1". We modified the Dijkstra algorithm to accommodate cases of completely unconnected nodes, by initializing the set of unvisited nodes with the neighbors of the starting node (instead of the whole PPIN) and augmenting this set each time with the neighbors of the next visited node. In this way, in case a node is unreachable from another, not all nodes of PPINs are visited.

Known MCL Drug Combinations: Table S1B catalogues combinatorial therapeutic strategies, including chemotherapeutic regiments collected from either the literature or the Drug Combination Database DCDB [7]. Starting from this information, we created a drug pair list of all possible combinations of the constituents of each regiment; we used this pair list as a golden truth to verify the drug combination analysis results.

Supplementary Table S2: SVM models trained for the separation of MCL stages.

	Stage	Kernel				
		degree-1	degree-2	degree-3	degree-4	Radial Basis
**TP Rate**	**N**	100.00%	93.50%	94.30%	83.75%	75.00%
	**IS**	100.00%	100.00%	100.00%	100.00%	75.00%
	**I**	100.00%	89.00%	85.25%	75.25%	75.00%
	**A**	100.00%	86.20%	84.00%	83.40%	100.00%
		100.00%	91.82%	90.59%	85.47%	82.35%
	**Stage**	**Classical stage sample prediction**				
**Prediction distribution**	**N**	0.00%	47.00%	0.00%	0.00%	0.00%
	**IS**	4.50%	51.50%	100.00%	100.00%	0.00%
	**I**	0.00%	0.00%	0.00%	0.00%	0.00%
	**A**	95.50%	1.50%	0.00%	0.00%	100.00%

Supplementary Table S3: Predicting Classical Stage samples using linear SVM classifier.

	Stage	Classical Stage Sample ID			
		GSM747375	GSM747376	GSM747377	GSM747378
**Prediction distribution**	N	0.00%	0.00%	0.00%	0.00%
	IS	0.00%	0.00%	0.00%	18.00%
	I	0.00%	0.00%	0.00%	0.00%
	A	100.00%	100.00%	100.00%	82.00%

Supplementary Discussion

Wnt signaling pathway

One of the foremost signaling pathway discussed in the original research paper of the GSE30189 dataset, as being related to MCL disease, is Wnt signaling pathway that is known to promote cell growth, orchestrated by APC protein, which prevents excessive cell growth and tumor formation. In the majority of colon cancers, mutations on APC gene lead to hyperactivation of the Wnt pathway and the appearance of cancer [8]. In MCL *in situ,* Wnt signaling is active as confirmed by immunohistochemistry, where Wnt3 is accumulated in the cytoplasm, and phosphorylated β-catenin in the nucleus [9]. Leukemia forms, like chronic lymphocytic leukemia (CLL) and T-cell ALL (T-ALL) seem to also dependent on active Wnt signaling for survival [10].

Three Wnt alternative signaling pathways exist, the canonical beta-catenin, the non-canonical cell polarity, and the Ca^{+2}. Nineteen human Wnt genes are known, which include additional alternative splicing variants. Wnt signaling is triggered by the binding of a Wnt protein to a Frizzled receptor (ten Frizzled receptors have been identified in human). Signaling specificity is determined through specific expression of these receptors, the formation of homo- and hetero- oligomers, and the participation of LRP coreceptors [10]. In the absence of Wnt signaling, β-catenin is marked for degradation by the destruction complex, which contains the scaffold protein Axin, APC and the kinases GSK3β and casein kinase (CK1α), and is maintained at low levels [10]. In the absence of nuclear β-catenin, a complex, containing T-cell factor/lymphoid enhancer factor (TCF/LEF) and transducing-like enhancer protein (TLE/Groucho), recruits HDACs to repress target genes. Upon activation of Wnt signaling, the deconstruction complex is inactivated, β-catenin translocates into the nucleus, and forms an active complex with TCF/LEF by displacing TLE/Groucho proteins, leading to the transcription of target genes, including CCND1 [11].

Salvador–Warts–Hippo signaling pathway

The Hippo pathway is a complex tumor suppressor mechanism that controls tissue growth and cell fate [12,13] and its deactivation is reported in pancreatic cancer [14] and breast cancer [15]. The main role of Hippo pathway is to prevent the YAP and TAZ proteins from getting translocated to the nucleus, where they induce the transcription of cell proliferation, survival, and stem cell maintenance genes. When active, the Hippo core complex phosphorylates the main effectors, YAP and TAZ, retaining them in the cytoplasm, where they undergo proteasomal degradation. E-cadherin (CDH1), a calcium-dependent cell adhesion protein, is one of the ligand-receptor complexes that is used by the Hippo pathway. Down-regulation of E-cadherin has been proposed for Hippo deactivation, and has been found to highly correlate with the hypermethylation of its promoter [14,15]. In relation to the above, activation of the Hedgehog pathway, enriched during the first MCL stage, leads to increased levels of the Snail protein, and decreased amounts of E-cadherin [16]. Furthermore, flow cytometry experiments demonstrate that activated Hippo pathway leads to Natural Killer T–Cell apoptosis [17]. Hippo and Wnt signaling pathways are related at the level of the destruction complex mentioned above, where bound YAP/TAZ are identified as Wnt negative transcriptional regulators of Wnt signaling pathway[10].

The comparative analysis of the GSE30189 dataset captures the downregulation of E-cadherin (cadherin-1 or CDH1) and up-regulation of F-actin (ACTB) (ItoA and BtoMCL comparisons respectively), which can also regulate the main effectors of the Hippo pathway (YAP, TAZ) through its polymerization. In cases like late-stage MCL patients, E-cadherin was found upregulated. YAP's and TAZ's role in the tumor microenvironment is crucial; they are functioning as hubs in the tumor signaling network, by orchestrating tumour-stromal interactions [18]. In our analysis, the Hippo pathway was found to be enriched at the *in-situ* stage and throughout the following stages of MCL (**Figure 2B, Table S5A**).

Hedgehog signaling pathway

Hedgehog signaling is regulating E-cadherin expression, to maintain the actin cytoskeleton (F-actin cortical expression) and tight junctions [19,20]. In RK3E cells, the Snail protein of the Hedgehog pathway was found to repress E-cadherin [16]. In our results, the Hedgehog pathway was found initially affected at the *in-situ* stage (potentially, due to CCND1 overexpression), and the Hippo pathway remained enriched, from the classical until the aggressive stage (**Figure 2B**). Another protein of interest is casein kinase I (CK1) of the Hedgehog pathway, an essential kinase that regulates cellular growth CK1 (delta isoform) and phosphorylates p53 at the N-terminal region, preferably at S20 [21] and topoisomerase IIalpha [22]. The delta isoform of CK1 was found to be down-regulated at the ItoA transition. Down-regulated CK1δ has been shown to result in accumulated MDM2, decreasing p53 activity and apoptosis induction after DNA damage [23,24].

BCR signaling pathway

The B-cell receptor signaling pathway (BCR) is crucial for the survival of B-cells, since it regulates B-cell fate (apoptosis, proliferation, and differentiation into memory B cells) through signal transduction and presentation of

peptides to helper T cells [25]. Key players of this regulatory pathway, such as Bruton's tyrosine kinase (BTK), phosphoinositide-3-kinase (PI3K), spleen tyrosine kinase (SYK) and BCL-2 have been the targets for the treatment of B-cell lymphomas. Kinase inhibitors have been developed aiming to regulate the BCR pathway, which remains active in B cell malignancies. A recently added arrow in the quiver is ibrutinib, a multi-kinase inhibitor with many toxicities. Ibrutinib is targeting BTK and other kinases, including tec family kinases (TFK)s, like TEC, TXK/RLK, ITK [26]. Apart from ibrutinib, other kinase inhibitors, including acalabrutinib, idelalisib, copanlisib, umbralisib, duvelisib and fostamatinib have been included in ongoing clinical trials for the treatment of R/R lymphoma [26,27]. In current analysis, "BCR signaling pathway" was found to be enriched and affected during intermediate and aggressive stages (**Table S5A**), with BTK downregulated at the aggressive stage, SYK up- and down-regulated and TXK/RLK down- and up- regulated at the progressed stage MCL patients (BtoMCL and MCL(e)toMCL), respectively.

One of the agents that caught our attention was fostamatinib, a newly approved drug originally used for the treatment of rheumatoid arthritis, with ongoing clinical trials for relapsed lymphoma [27]. Fostamatinib was found to have only 11% response rate in patients with relapsed MCL. Its known mechanism of action is the inhibition of the tyrosine-protein kinase (SYK) of the B-cell receptor (BCR) pathway [27]. Fostamatinib stood out in our drug repurposing analysis as a "hub" drug, targeting genes in three of the four MCL stages, among them Bruton tyrosine kinases (BTK), MAP2K2, PTK2B (**Figure 4,** cyan rhombus). The importance of BTK gene in the BCR pathway has already been acknowledged, and current MCL regiments have included ibrutinib, a potent covalent kinase inhibitor that targets BTK (**Table S1B**). PTK2B is another tyrosine kinase, required for normal levels of marginal zone B-cells in the spleen [28] and normal migration of splenic B-cells. Moreover, PTK2B has a role in P53/TP53 proteasomal degradation, by recruiting the ubiquitin ligase MDM2 to P53/TP53 in the nucleus.

AGE-RAGE signaling pathway in diabetic complications

AGE-RAGE signaling pathway in diabetic complications was one of the pathways detected only by pathfindR analysis (*in situ* stage; **Table S3A, C**). AGEs (advanced glycation end products) are produced non-enzymatically after the reduction of sugars and proteins during aging or other conditions. AGEs interact with RAGE, their receptor and activate pathway signal transduction. AGE-RAGE signaling is considerably influencing apoptosis, autophagy, and necroptosis in the cancer cells [29]. The receptor for advanced glycation end-products RAGE is overexpressed in a variety of cancer types including pancreatic, gastric, breast, lung cancers and lymphoma [30,31]. RAGE ligands are secreted by cancer cells within the tumor microenvironment and stimulate metastasis and proliferation [31].

VEGFA and SPARC potential role in MLC tumor growth and progression

Vascular endothelial growth factor-A (VEGFA), an important angiogenic molecule and the main stimulator of endothelial cell proliferation, plays an important role on tumor angiogenesis [32]. In solid tumors, VEGFA overexpression is associated with increased angiogenesis, tumor growth, and metastasis [33].

Lymphoma associated angiogenesis has similar features, involves direct production of proangiogenic factors (like VEGFA) by lymphoma cells, and interactions between lymphoma cells and the tumor microenvironment (TME). Activated B cells secrete VEGFA, thus they have an angiogenic potential [34]. Immunohistochemistry on archival specimens of CD5+ MCL patients verifies VEGFA overexpression [35]. In human MCL cell lines, it was also shown that upon treatment with HDAC inhibitors, VEGFA production is shut down and growth suppression and apoptosis is induced, while cyclin D1 is downregulated [36]. VEGFA is also detected in aggressive DLBCL and indolent CLL nodes [34]. In angioimmunoblastic T-cell lymphoma (AITL), increased levels of VEGFA gene expression both in lymphoma cells and endothelial cells, were related to extranodal involvement and to short survival time. VEGFA protein and its receptor VEGF-R1 were coexpressed on endothelial cells of microvessels in the areas of lymphoma invasion [32]. In B cell non-Hodgkin lymphoma (B-NHL) certain angiogenic parameters were identified as prognostic markers, also VEGFA antibodies and receptor tyrosine kinase inhibitors targeting VEGF receptors have been considered for therapy [34]. Lenalidomide, an immunomodulatory drug, inhibits angiogenesis and proliferation in B cell non-Hodgkin lymphomas (NHL), with greatest potency against MCL. More specifically, SPARC mRNA expression was found to be significantly correlated with both the anti-proliferative and the VEGF-suppressive effects of lenalidomide on MCL cells [37].

SPARC (secreted protein acidic and rich in cysteine), otherwise osteonectin or basement membrane (BM 40), is a matricellular non-structural glycoprotein that binds to Ca^{2+} or collagen. Matricellular proteins are multifunctional proteins that participate in a diversity of functions, by interacting with structural extracellular matrix molecules, cell surface receptors, proteases, and growth factors. SPARC's known functions are epithelial–mesenchymal transition, immune surveillance, angiogenesis and counteradhesion and antiproliferation of endothelial cells [38]. The antitumor effect of SPARC is not completely understood; however, it is known that SPARC plays a significant role in altering

cancer cell activity and the tumor's microenvironment (TME) and in promoting metastasis [39,40]. The expression levels of SPARC correlate with tumor progression and migration, but the manner depends on tissue and tumor cell type [39,40]. For example, in prostate and breast cancer and glioma there is a negative relation between high levels of SPARC and tumor growth and cell proliferation, whereas in melanoma is associated with aggressiveness [39,40]. In medulloblastoma SPARC high levels correlate with six miRNAs involved in cancer progression [41]. SPARC inhibits VEGF- and fibroblast growth factor-2-stimulated proliferation of endothelial cells [39,40]. Nab-paclitaxel (nanoparticle albumin bound paclitaxel), a chemotherapeutic agent used in various cancers, is a potential drug that associates with SPARC since it may increase efficacy in TME [40].

Here, we have identified SPARC and VEGF-A being overexpressed in late- versus early- stage MCL (MCL(e)toMCL; **Table 1, S4**) and the related pathways "miR-509-3p alteration of YAP1/E" and the "VEGF signaling" pathways respectively" as being enriched. VEGF-A's role in the late-stage MCL is further supported by the results of co-expression functional module analysis, where VEGF-A is included in the network of the common module between health B cells and late MCL patients (**Figure S8A**). Paclitaxel is proposed, by our analysis, for the treatment of early MCL, mainly because of its target TUBB1 (**Table S6B**).

Oxidative phosphorylation

Tumor cell survival and resistance to chemotherapy requires oxidative phosphorylation, in certain cancer types, such as high grade serous ovarian cancer [42]. Recent published data find evidence that MCL relapse after initial treatment with ibrutinib, can be attributed to a metabolic reprogramming and a shift toward reliance on oxidative phosphorylation [43]. Furthermore, switching from aerobic glycolysis to oxidative phosphorylation in mitochondria, both in chronic lymphocytic leukemia (CLL) and MCL cells, modulates sensitivity to apoptosis induced by TRAIL (TNF (tumor necrosis factor)-related apoptosis-inducing ligand). Therefore, in relapsed/refractory cancers, targeting dysregulated metabolic pathways may overcome resistance.

In current analysis, KEGG pathways related to metabolism were enriched in all MCL stages except for *in situ*, these include "Oxidative phosphorylation", "Citrate cycle (TCA cycle)", "Fructose and mannose metabolism", "Propanoate metabolism", "Biosynthesis of unsaturated fatty acids", "Purine metabolism", "Central carbon metabolism in cancer", "Choline metabolism in cancer" and "Non-alcoholic fatty liver disease". Also, NADH and ATP energy carriers were selected in the drug repurposing analysis step, together with several nutraceuticals such as calcium, citric acid, different forms of vitamin E etc. (**Table S6**).

Non-coding transcripts in cancer

Our pipeline has identified 1011 deregulated ncRNA at the different MCL stages; for several of them, the correlation with tumor outcome, progression, stage, and metastasis has been tested in clinical and experimental studies [44]. Three types of ncRNA were measured in the MCL transcriptomic datasets, these included lncRNAs (long noncoding RNAs), asRNAs (antisense RNAs), small conditional RNA (scRNA), snRNAs (small nuclear RNAs) and small nucleolar RNAs (snoRNAs), with the later known to act as regulatory molecules that mediate cancer processes through alternative splicing [45]. In the following paragraphs, we discuss selected ncRNA detected in our analysis that are potentially involved in MCL progression.

Antisense RNA IGF2BP2-AS1 was found downregulated and its target IGF2BP2 (overexpressed in late-stage MCL) promotes tumor progression in a variety of cancers, such as glioblastoma multiforme (GBM) and gallbladder cancer [46,47]. Further studies have showed that IGF2BP2 regulates the translation of insulin-like growth factor 2 (IGF2) and increases the PI3K/Akt signaling pathway activation [48].

Long non-coding RNA 91H was found overexpressed in late-stage MCL patients (BtoMCL and MCL(e)toMCL) compared to healthy people or newly diagnosed. LncRNA H19 (otherwise known as 91H) regulates IGF2 expression by interacting with IGF2BP2, as verified by immunoprecipitation assay [49]. H19 is found located in an imprinted region of chromosome 11 near IGF2 gene. Mutations in this gene have been associated with Beckwith-Wiedemann Syndrome and Wilms tumorigenesis [49], whereas H19's high expression has been related to high risk in tumor metastasis in colorectal cancer [49].

UNC5B antisense RNA 1 was found to be overexpressed in late-stage MCL patients and was included in the respective common module (**Figure S8A**). The target of UNC5B-AS1, UNC5B is a netrin receptor required for axon guidance, which in the absence of netrin, mediates apoptosis by activating death-associated-protein kinase (DAPK)[50]. Recent study illustrates that UNC5B-AS1 overexpression promotes the progression of ovarian cancer via a mechanism that involves interaction with zeste 2 polycomb repressive complex 2 subunit (EZH2), triggering histone H3 trimethylation and epigenetic repression of N-myc downstream regulated gene-2 (NDRG2) [51]. UNC5B-

AS1 is significantly upregulated in metastatic papillary thyroid cancer (PTC) and its downregulation via siRNA leads to inhibition of cell proliferation, colony formation, migration, and invasion of PTC cell lines [52].

LncRNA CBR3-AS1 is upregulated in newly diagnosed MCL patients. Increased expression of CBR3-AS1 in breast cancer tissues is closely correlated with poor prognosis and mediates chemotherapy resistance via a mechanism that involves miR-25-3p and MEK4/JNK1 regulatory axes [53]. Another antisense RNA that was found significantly upregulated in late-stage versus newly diagnosed MCL is LBX2-AS1. In hepatocellular carcinoma [54], upregulation of LBX2-AS1 is significantly correlated with shorter overall survival. Functional studies in hepatocellular cells suggest that LBX2-AS1 competes microRNA-384 (miR-384) and that knockdown of LBX2-AS1 reduces proliferation and induces *in vitro* apoptosis [54].

Fifty-nine long noncoding RNAs were found deregulated in MCL, seven in intermediate and aggressive stages, thirty-seven in late-stage MCL patients and twenty-one in newly diagnosed patients (thirteen of them common). Below, we discuss two examples of lncRNAs that were identified by common functional module analysis in MCL sample groups. Over-expression of LINC01139 is observed in advanced Tumor, Node, Metastasis (TNM) stage and has poor prognosis in hepatocellular carcinoma (HCC), while its knockdown has been found to suppress cell proliferation [55]. Down-regulated LINC01003, which is a member of the common module between late-stage MCL, and healthy B-cells, is also observed at low expression levels in MM cell lines and peripheral blood samples of MM patients [56]. Up-regulation of LINC01003 represses cell viability and adhesion, promotes apoptosis of MM cells and suppresses xenograft tumor in mice via down-regulation of miR-33a-5p, a downstream target of LINC01003 [56]. Finally, two lncRNAs LINC00597 and LINC00112 were is also differentially expressed in NSCLC Tumors [57] and gastric cancer [58].

Proposed biomarkers, drugs, and targets

The comparative analysis has highlighted genes with differential expression at MCL stages compared to other stages or healthy cases. However, among these genes we found false positives or genes not directly related to the molecular effect of the (IgH/CCND1) chromosomal translocation. Functional co-expression module discovery attempts to contribute with coherent gene transcription (i.e., with potential cause and effect relations). Theses analyses, together with drug repurposing and drug combination prediction, have pointed out genes of potential diagnostic and therapeutic value. In the following paragraphs, we discuss in more detail existing experimental or clinical evidence for some of the genes identified by our analysis and for several of the compounds targeting them, and their potential role as biomarkers or treatments for MCL.

Serotonin receptor 2A (HTR2A) is molecule of interest that was found up-regulated in the intermediate and aggressive MCL stages. Fifty-five drugs treating mental illnesses (e.g., psychosis), were selected in the drug-repurposing analysis for targeting HTR2A. Increased levels of serotonin (5-HT) and serotonin receptor levels may be relevant to cancer progression. More than half of the cancer patients suffer also from symptoms of depression, and selective serotonin reuptake inhibitor (SSRI) is frequently prescribed as an anti-depressant [59]. A recent analysis demonstrated that serotonin receptor mRNAs and proteins are expressed across diverse cancer types, and that pharmacological inhibition of 5-HT receptors may lead to activation of the p53 DNA damage pathway and suppression of MAPK activity. It was also discovered that SSRI anti-depressant medications is associated with more than 2-fold increase in tumor proliferation rates, suggesting that serotonin signaling pathways promote cell growth and survival [60].

Our analysis finds that polyunsaturated fatty acid 5-lipoxygenase (ALOX5) is up-regulated in aggressive and intermediate stages, also both in early- and late-stage MCL. Cells primarily involved in regulating inflammation, allergy, and other immune responses, e.g., neutrophils, eosinophils, basophils, monocytes, macrophages, mast cells, dendritic cells, and B-lymphocytes, express ALOX5. Platelets, T cells, and erythrocytes are ALOX5-negative. ALOX5 products, particularly 5-hydroxyeicosatetraenoic acid and 5-oxo-eicosatetraenoic acid, promote the proliferation of these ALOX5 aberrantly expressing tumor cell lines, suggesting that ALOX5 acts as a pro-malignancy factor for them, and by extension for their parent tumors [61]. Certain peptide-leukotrienes of these have shown to promote the growth of cultured human breast cancer and chronic lymphocytic leukemia cell lines, thereby suggesting that ALOX5 may contribute to the progression of these diseases. Studies implicate ALOX5 in contributing to innate immunity, by adding to the mounting of inflammatory responses to a wide range of acute responses (e.g., pathogen invasion, trauma, and burns); however, ALOX5, also, contributes to the development and progression of excessive and chronic inflammatory responses, such as rheumatoid arthritis, atherosclerosis, inflammatory bowel disease, and autoimmune diseases.

One of the few drug targets for the initial MCL stage was estrogen receptor beta ERβ. The estrogen-signaling is mediated by ERα and ERβ receptors, and there is evidence that imbalanced ERβ expression is related with tumor development and progression [64]. In lymphoma, vascularization and dissemination is prevented by estradiol and other estrogen receptor β agonists in xenograft experiments [64]. Another target was 5-LO, a pro-inflammatory enzyme, expressed in immune and cancer cells [65], with its role in tumor microenvironment still unclear. A recent study suggests that 5-LO is suppressed in apoptotic cancer cells via MerTK[65], possibly marking (tumor-associated macrophages) TAM polarization; however, in our analysis, 5-LO was found rather overexpressed.

A potential MCL biomarker is the beta subunit of Hemoglobin (HBB), which was found to be significantly up-regulated at the classical and aggressive stages. Recently HBB has been proposed as a biomarker for breast cancer [62]. Cells that overexpress HBB acquire a more migratory and invasive phenotype, and this is reversed by blocking its oxygen-binding site [62,63].

One of the agents that caught our attention was fostamatinib, a newly approved drug originally used for the treatment of rheumatoid arthritis, with ongoing clinical trials for relapsed lymphoma [27] Fostamatinib was found to have only 11% response rate in patients with relapsed MCL. Its known mechanism of action is the inhibition of the tyrosine-protein kinase (SYK) of the B-cell receptor (BCR) pathway [27]. Fostamatinib stood out in our drug repurposing analysis as a "hub" drug, targeting several genes, in three out of the four MCL stages, such as Bruton tyrosine (BTK), MAP2K2, PTK2B kinases (**Figure 4,** cyan rhombus). The importance of BTK gene in the BCR pathway has already been acknowledged, and currently regiments for the treatment of MCL include ibrutinib, a potent covalent kinase inhibitor that targets BTK (**Table S1B**). PTK2B is another tyrosine kinase, required for normal levels of marginal zone B-cells in the spleen [28] and normal migration of splenic B-cells. Moreover, PTK2B has a role in p53/TP53 proteasomal degradation, by recruiting the ubiquitin ligase MDM2 to p53/TP53 in the nucleus.

Arsenic trioxide, the only inhibitor of the CCND1 gene during the *in situ* MCL stage, has been recently found to have antitumor effect in MCL cell lines [66]. Aldesleukin, a drug proposed for the intermediate MCL stage agonist of the IL2RG cytokine receptor, has terminated the second phase of clinical trials and is tested in combination with rituximab for Non-Hodgkin's Lymphoma (NHL) treatment.

Our analysis revealed a significant number of antiphycotic drugs proposed for the treatment of MCL at the most progressed stages (Intermediate, Aggressive, **Figure 4, S10B**). Interestingly, a recent review mentions that patients treated for schizophrenia have lower incidences of certain types of cancer, such as respiratory, prostate, and bladder cancers [67]. Antipsychotic drugs induce apoptosis and suppress metastasis in *in vitro* and *in vivo* models through mechanisms involving p53, STAT3, STAT5, protein phosphatase 2A, cholesterol homeostasis, integrins, autophagy, USP1, wnt/β-catenin signaling, and DNA repair. Specifically, fluspirilene was shown to inhibit the p53-MDM2 interaction by binding to the p53-binding pocket of the MDM2 protein, resulting in p53 activation and inhibition of human colon cancer cell growth [67]. Another antipsychotic agent, penfluridol, was recently proven to have strong antitumour effect in various cancer cell lines, including breast, pancreatic, glioblastoma, and lung cancer cells [67,68].

Duvelisib, is a medication used to treat chronic lymphocytic leukemia (CLL), relapsed or refractory, small lymphocytic lymphoma (SLL). Clinical trials have concluded that Duvelisib [27] has response rates of 54% and 67%in CLL/SLL, and MCL/FL, correspondingly. Phosphatidylinositol 4,5-bisphosphate 3-kinase catalytic subunit gamma isoform (PIK3CG), is a target of both duvelisib and fostamatinib; it was significantly downregulated in both early- and late-stage MCL, although it was not included in the top proposed drugs. Finally, podofilox, proposed in all stages other than *in situ* (Table S6), is a nonalkaloid toxin lignan extracted from the roots and rhizomes of the Podophyllum species, with important antineoplastic and antiviral properties [69].

Supplemental Figures

<u>Supplementary Figure S1</u>: MDS analysis of the MCL stages (GSE30189) dataset

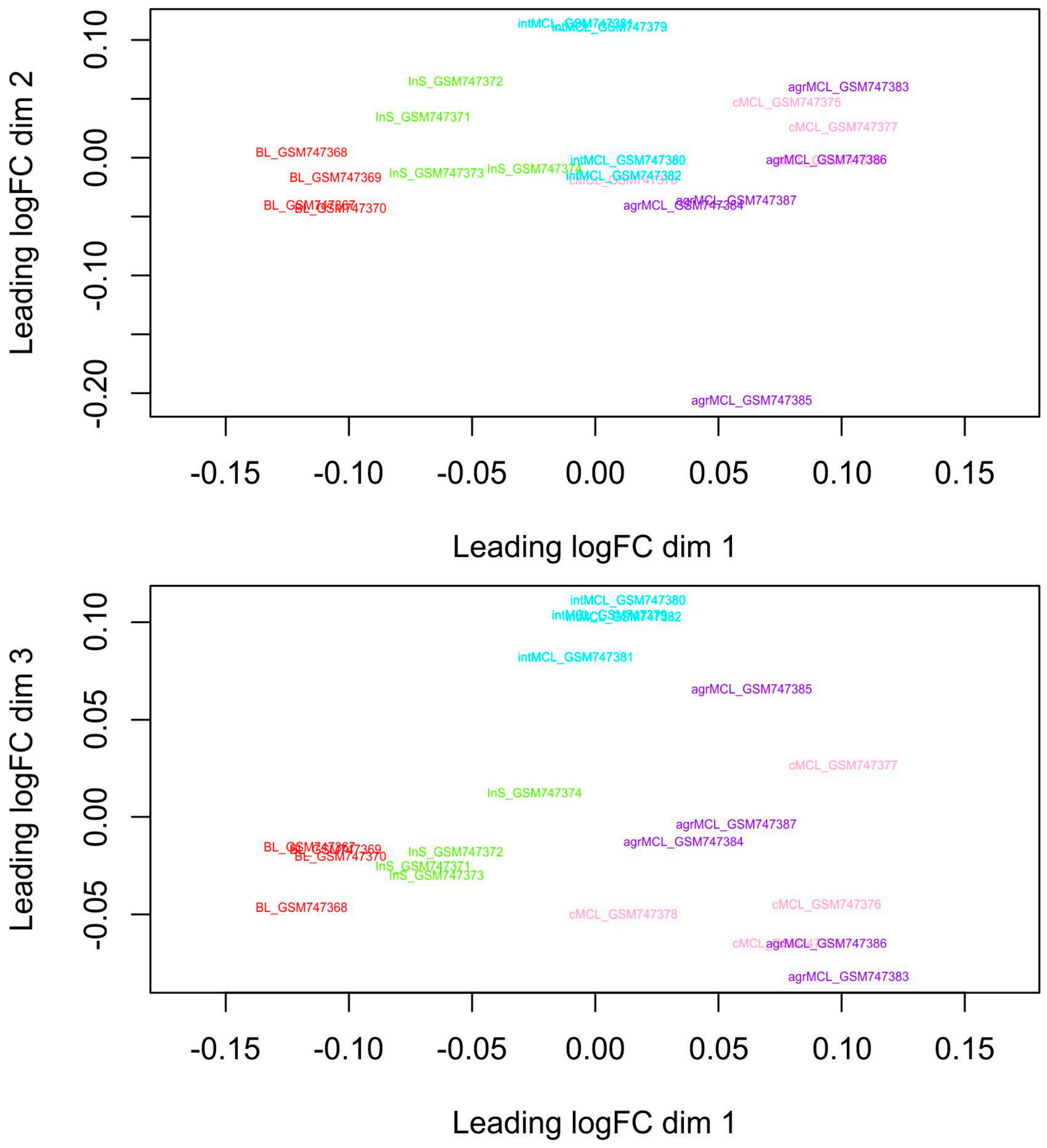

Pairwise scatter plots of the three leading dimensions of the MDS analysis for the GSE30189 dataset. The root-mean-square deviation was used as a distance metric, and the 500 most variable genes were considered (highest variance between all samples). The MDS analysis suggests that the normal (benign lymphadenitis, BL_GSM747367-70) and *in situ* stages (InS_GSM747371-74) are well separated considering the leading dimensions, whereas the classical stage samples (cMCL_GSM747375-78) seem to overlap with intermediate (intMCL_GSM747379-82) or aggressive stages (agrMCL_GSM747383-87). (BL: Benign Lymphadenitis; InS: In Situ MCL; intMCL: Intermediate MCL; cMCL: Classical MCL; argMCL: Aggressive MCL)

<u>Supplementary Figure S2</u>: MDS analysis of the MCL tissues (GSE45717) dataset

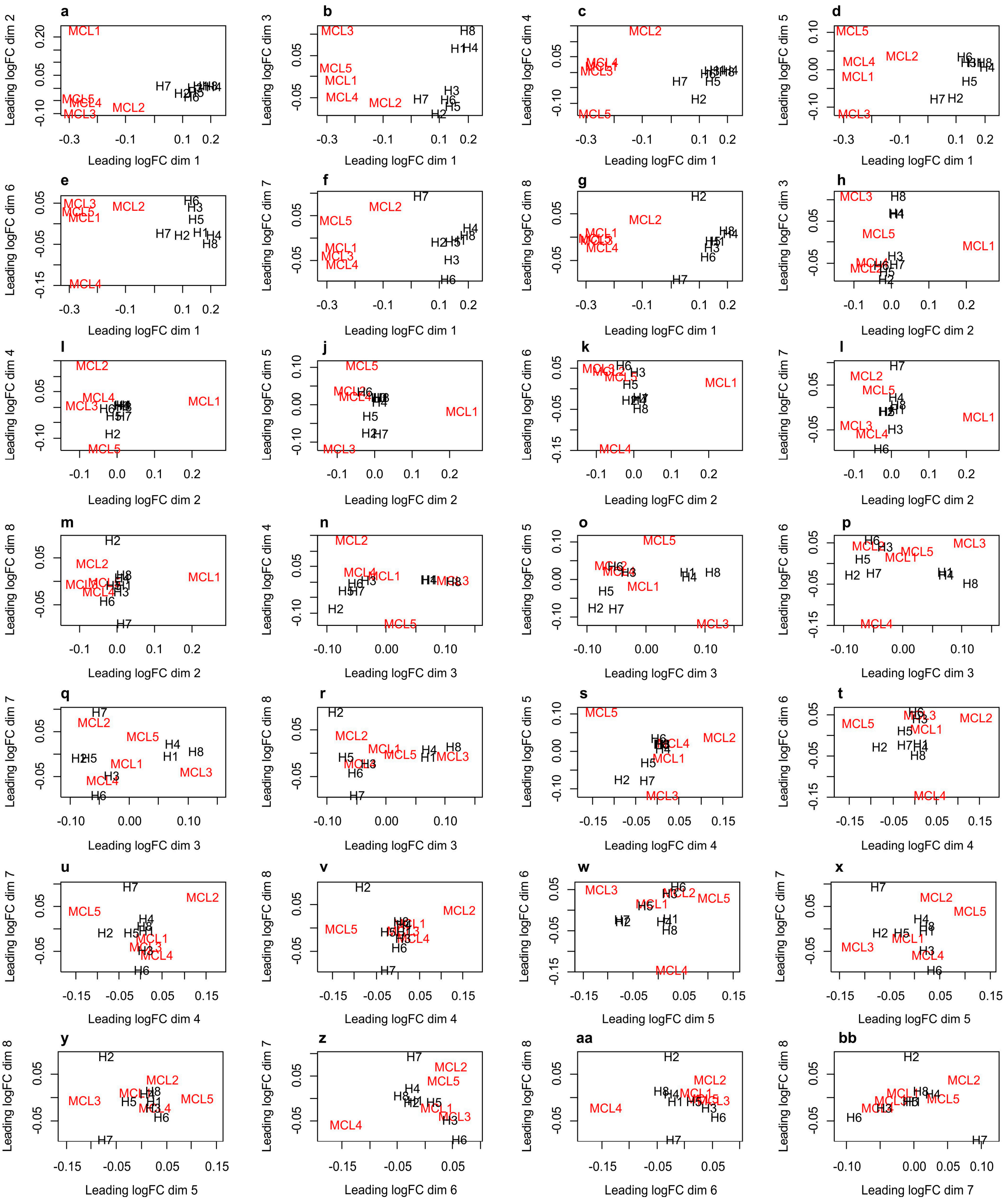

Pairwise scatter plots of the eight leading dimensions of the MDS analysis for the GSE45717 dataset. The root-mean-square deviation distance was calculated for the 500 most variable genes. The MDS plots highlight the heterogeneity of MCL transcriptional profiles compared to the healthy ones [i.e., tissue samples taken from healthy people (H1-7) are grouped together in several dimensions, whereas the MCL cases (MCL1-4) are more scattered].

Supplementary Figure S3: Affymetrix Human Genome U133 Plus 2.0 array data

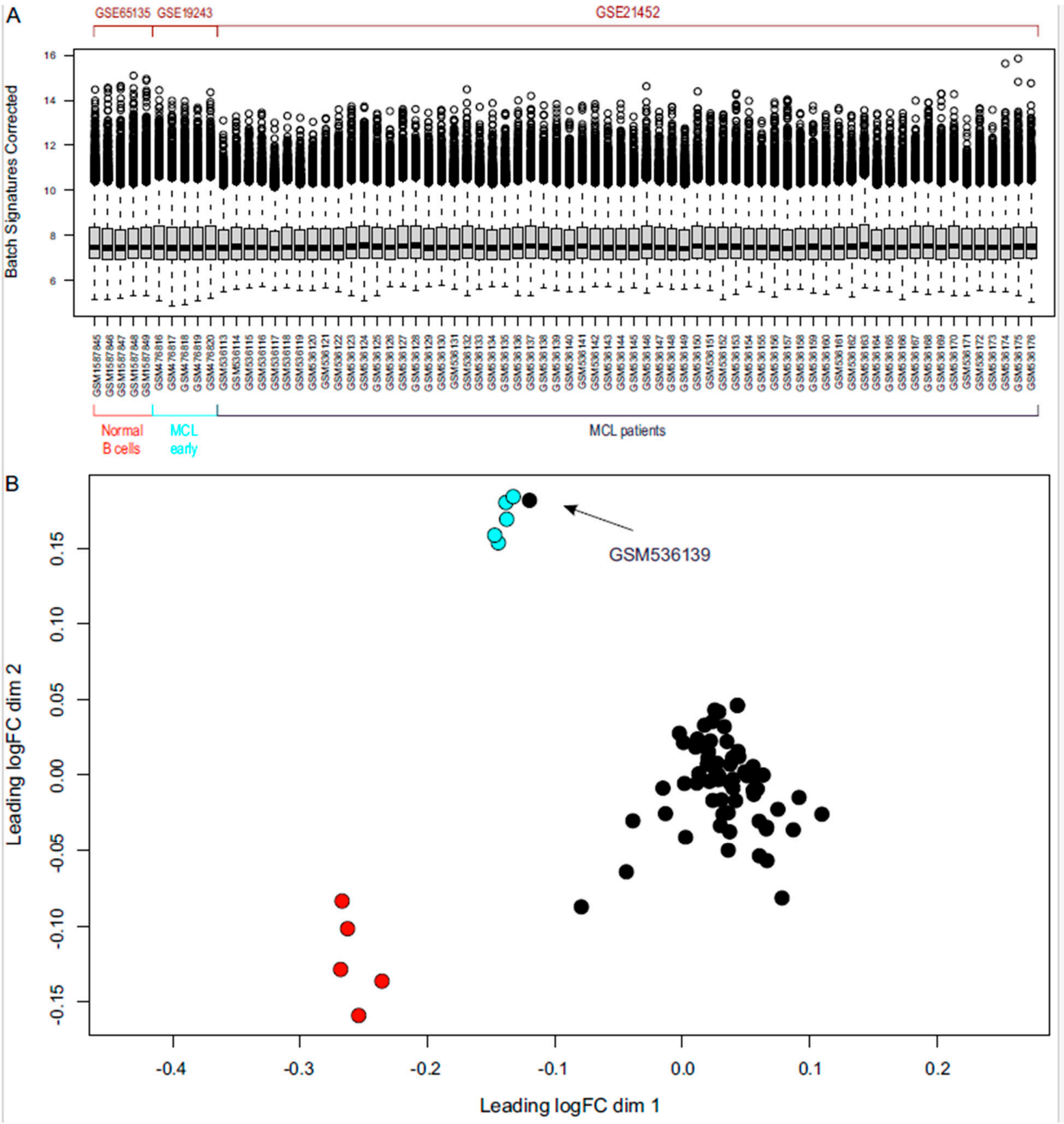

A. Boxplot and **B.** MDS analysis of the corrected RMA values of the top 100 variable genes. Corrected values are calculated after removing technical variation that is the result of batch effect signatures (see supplementary information above). We observe that one out of the 64 patient samples of the GSE21452 dataset (GSM5361139) is clustered together with the five newly diagnosed MCL patients of the GSE19243 dataset, suggesting that this patient is at early MCL stage.

Supplementary Figure S4: **Hierarchical clustering heatmaps of transcription profiles**

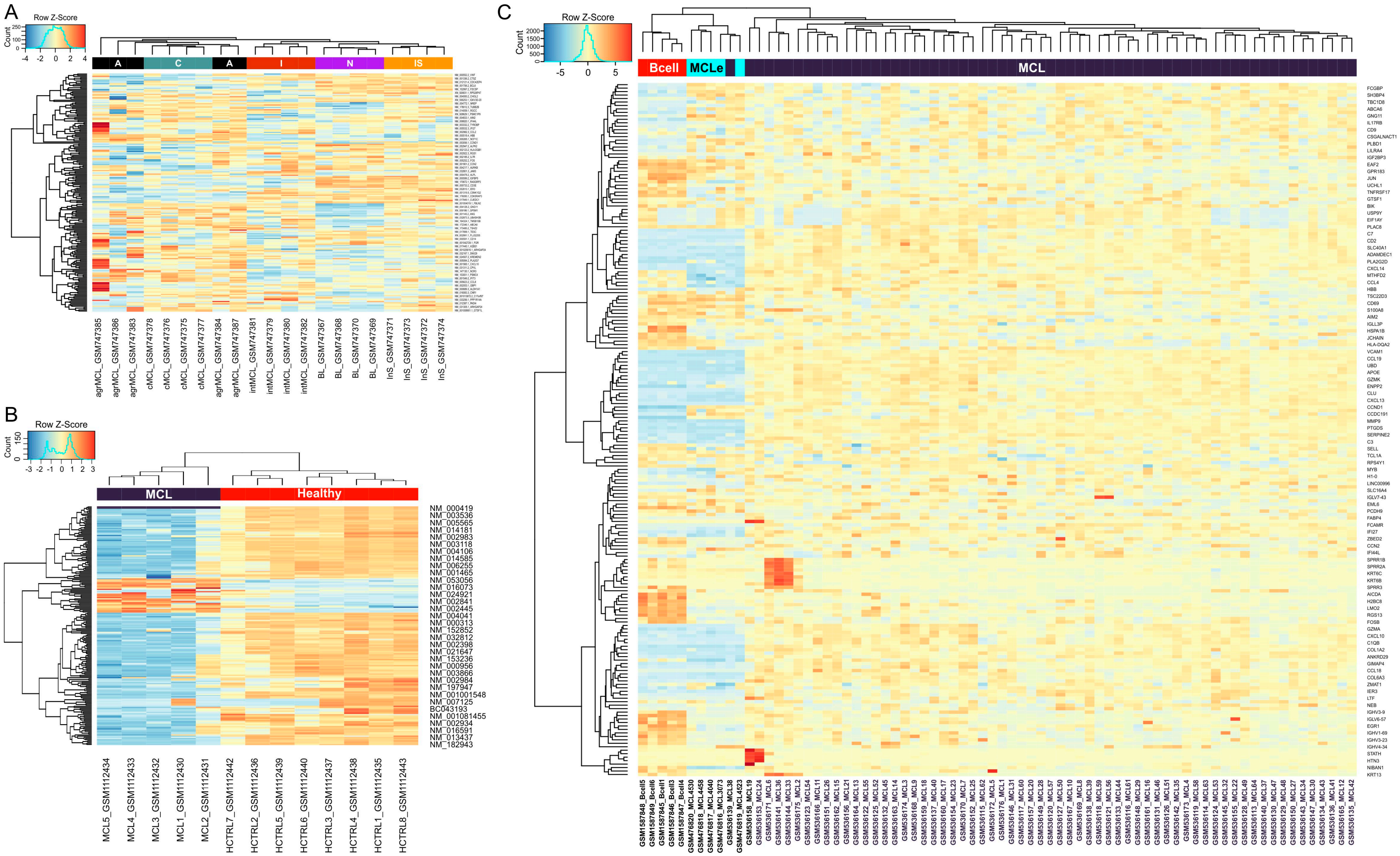

The 200 most variable genes are depicted in the form of hierarchical clustering heatmaps (z-score of the log2 counts per million) for: **A.** GSE30189 and **B.** GSE45717 **C.** GSE21452/GSE65135/GSE19243 datasets. We observe that tissue samples of the normal (N), *In situ* (IS) and intermediate (I) morphological groups, are clustered together by the hierarchical clustering. However, samples of the aggressive morphological group (GSM747384 and GSM747387) are clustered together with the classical (GSM747378) group. Together, classical, and aggressive morphological groups belong to the same second level branch of the hierarchical tree. Additionally, one MCL patient of the GSE21452 dataset (C) has similar profile with the five newly diagnosed MCL patients analyzed in GSE19243 dataset.

<u>Supplementary Figure S5</u> Analytic pipeline of MCL gene expression data analysis

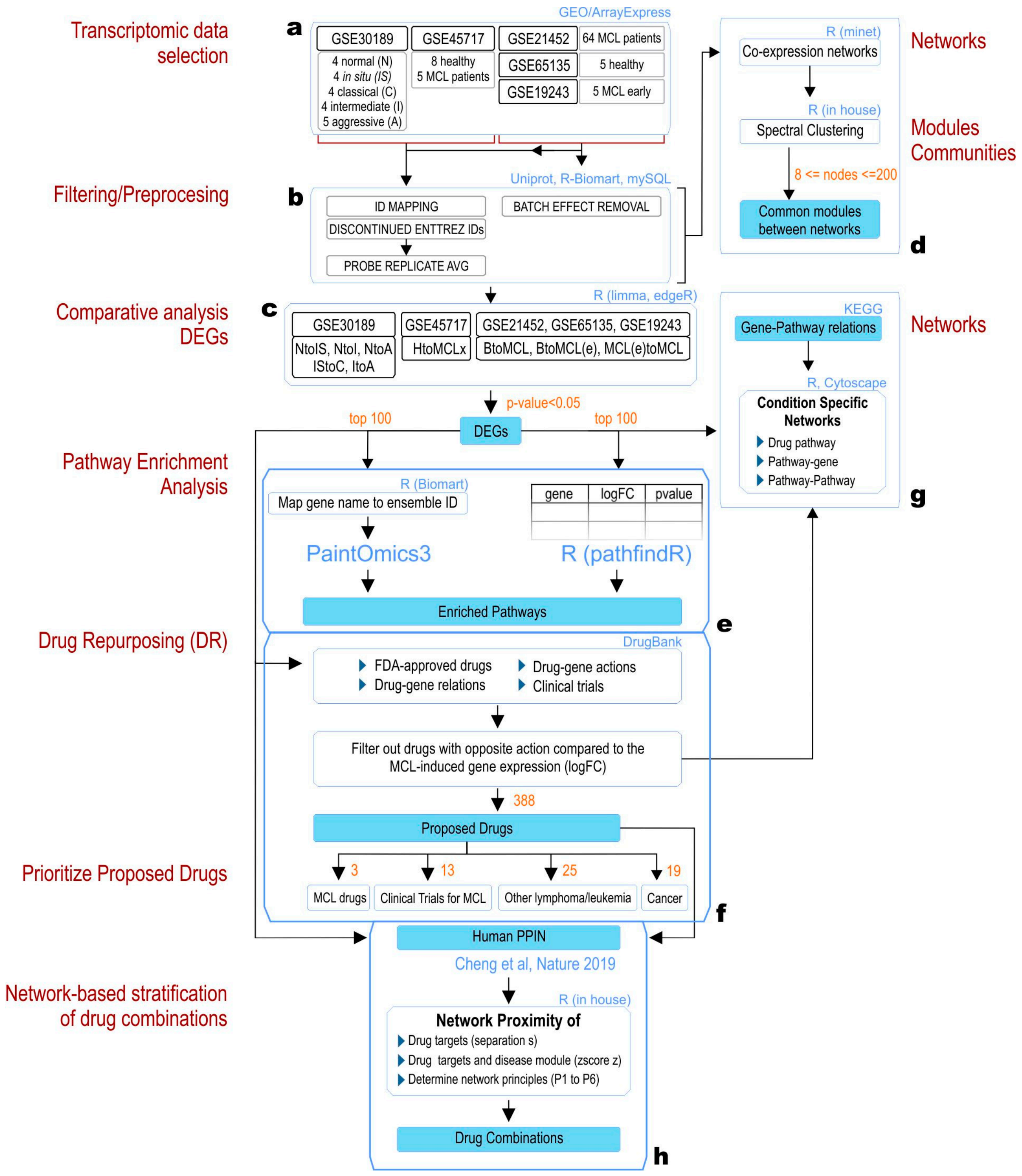

Detailed workflow of the bioinformatics analysis of the MCL transcriptomic data. As a first step, five transcriptomic datasets of MCL and healthy cases were selected (a). Next, microarray probes and their correspondence to Refseq or Entrez IS was reconsidered (b). Discontinued entrez IDs were excluded from the final transcript list, whereas in other cases the entrez ID was updated. Data were preprocessed using the batch effect removal method (where applicable). Processed transcriptomic data of valid/updated transcripts, proceeded to both 1) the comparative and 2) the gene co-expression analyses (c and d). The comparative analysis identified differentially expressed genes (DEGs) between different pairs sample groups, for example between MCL stages (NtoIS, IStoI, ItoA) or between

MLC patients at mixed-stages and healthy individuals. Next, the lists of DEGs were used as input for the three subsequent and independent analyses: the pathway enrichment (performed by PaintOmics and pathfingR tools; e), the drug repurposing (aided by Cytoscape; f) and the effective drug combination prediction analyses [6] (h). Alongside the comparative analysis, the processed transcriptomic profiles were used to construct co-expression networks (using the MRNETB algorithm); pairs of co-expression networks were analyzed using spectral clustering aiming to identify the co-expressed communities that are common between sample group pairs (d).

Supplementary Figure S6: Pathway-pathway networks at different MCL stages

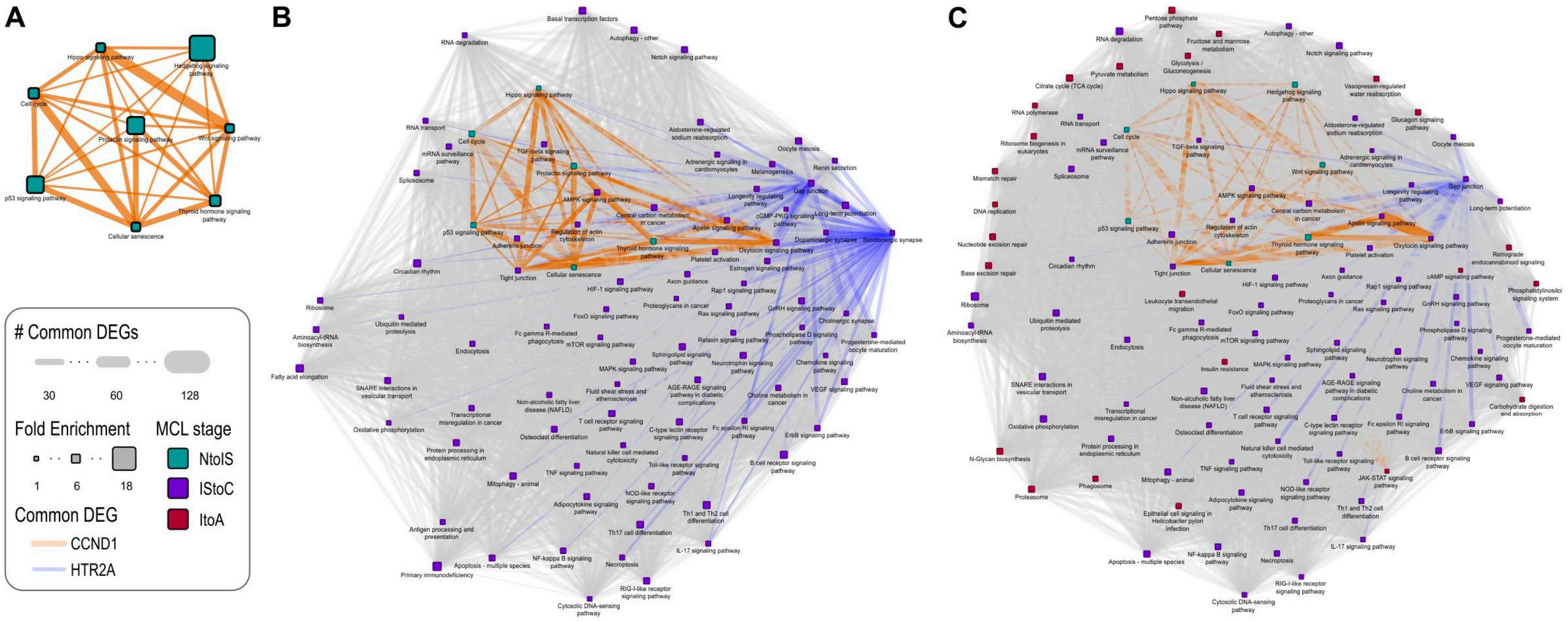

Pathway-pathway relations based on the common genes between enriched KEGG pathways at **A.** NtoIS, **B.** IStoI and **C.** ItoA MCL stage comparisons. Node size and edge weight is proportional to the fold enrichment value (pathfindR) and the number of common genes respectively (KEGG human pathways). Pathways are colored, based on the stage the respective pathway was first identified to be enriched (Teal: NtoIS, purple: IStoI and red: ItoA).

<u>Supplementary Figure S7:</u> Spectral clustering analysis – Eigenvalue Curves

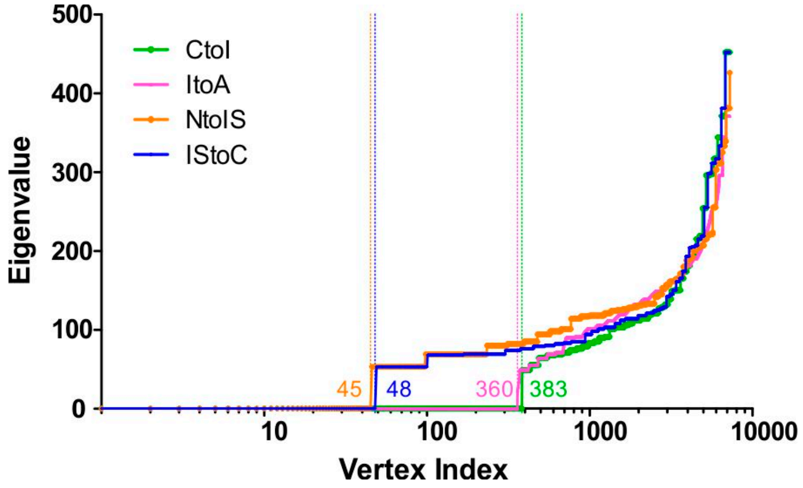

Eigenvalue curves of the respective *C* matrices constructed for every spectral clustering analysis that was performed. Spectral clustering compares the transcriptional co-expression networks of sample group pairs (e.g., NtoIS, BtoMCL; see Table 1) and identified common modules. The vertical lines indicate the eigenvalue gaps, i.e., the indicative number k of clusters for each spectral clustering [70]. In practice, smaller values k values than the ones suggested by the first eigenvalue gap were chosen. Spectral clustering into too many clusters (e.g., k>4000) leads to frequently disconnected and exceedingly small modules (< eight nodes per module: TableS8).

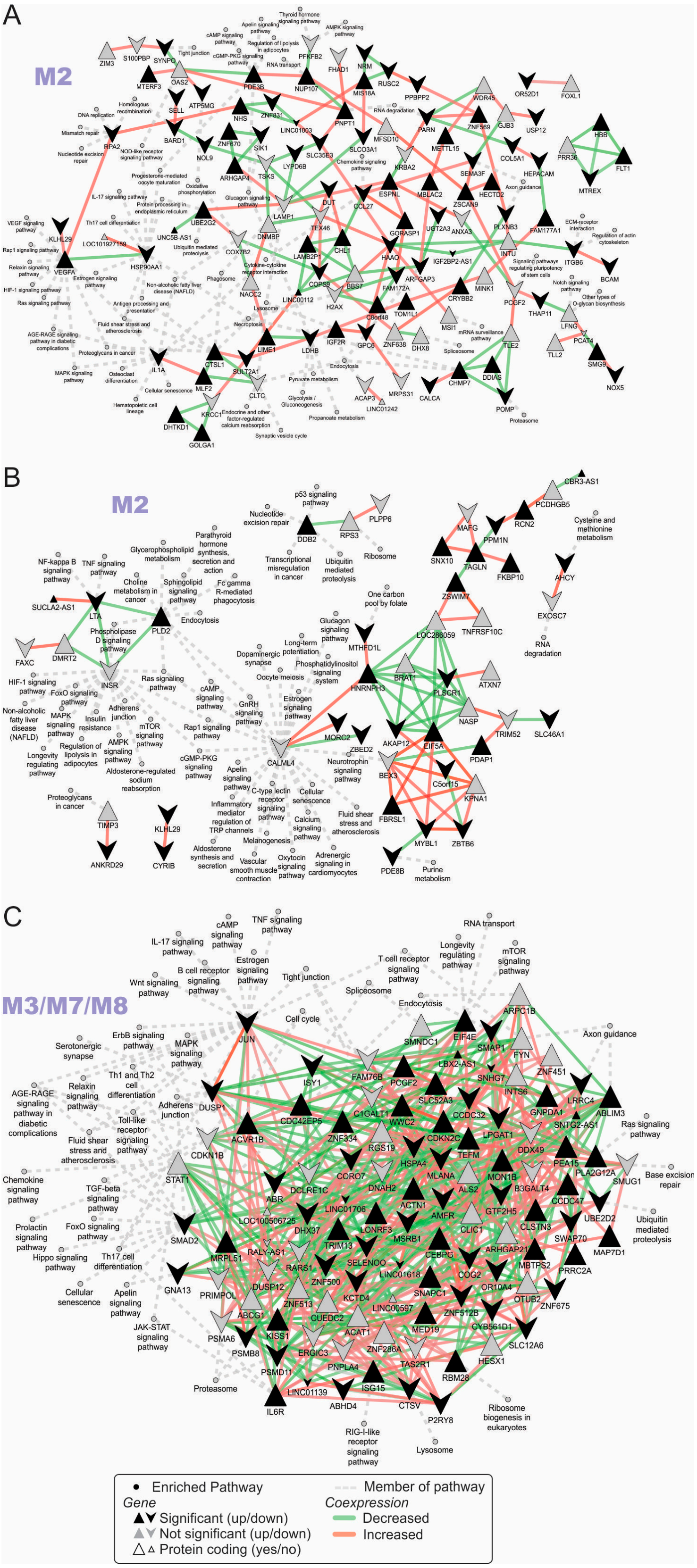

<u>Supplementary Figure S8:</u> Co-expression network and module alignment: Affymetrix U133 Plus 2.0 array data

Transcriptional co-expression networks of three sample groups were constructed using the MRNETB algorithm (Normal B cells, MCL newly diagnosed, MCL mixed stages) [71]. Modules of co-expressed genes were identified and aligned between networks using a spectral clustering approximation approach[70]. The union of the aligned modules between **A.** Normal B cells and MCL patients at progressed stage (BtoMCL) **B.** Normal B cells and MCL newly diagnosed patients (BtoMCL(e)) **C.** MCL patients at progressed stage and MCL newly diagnosed patients (MCLtoMCL(e)). Significantly differentially expressed genes are filled in black; up- and down- regulated genes are represented as regular and flipped arrowheads correspondingly, whereas enriched pathways are depicted as grey circles. NcRNAs are depicted in smaller arrowhead sizes. The alteration in co-expression (i.e., $\log_2$ fold change) is used as a weight of the connecting edges. Loss of co-expression is depicted with green (negative $\log_2$ fold change), whereas gain of co-expression with red (positive $\log_2$ fold change).

<u>Supplementary Figure S9:</u> Summarizing proposed MCL drug's features

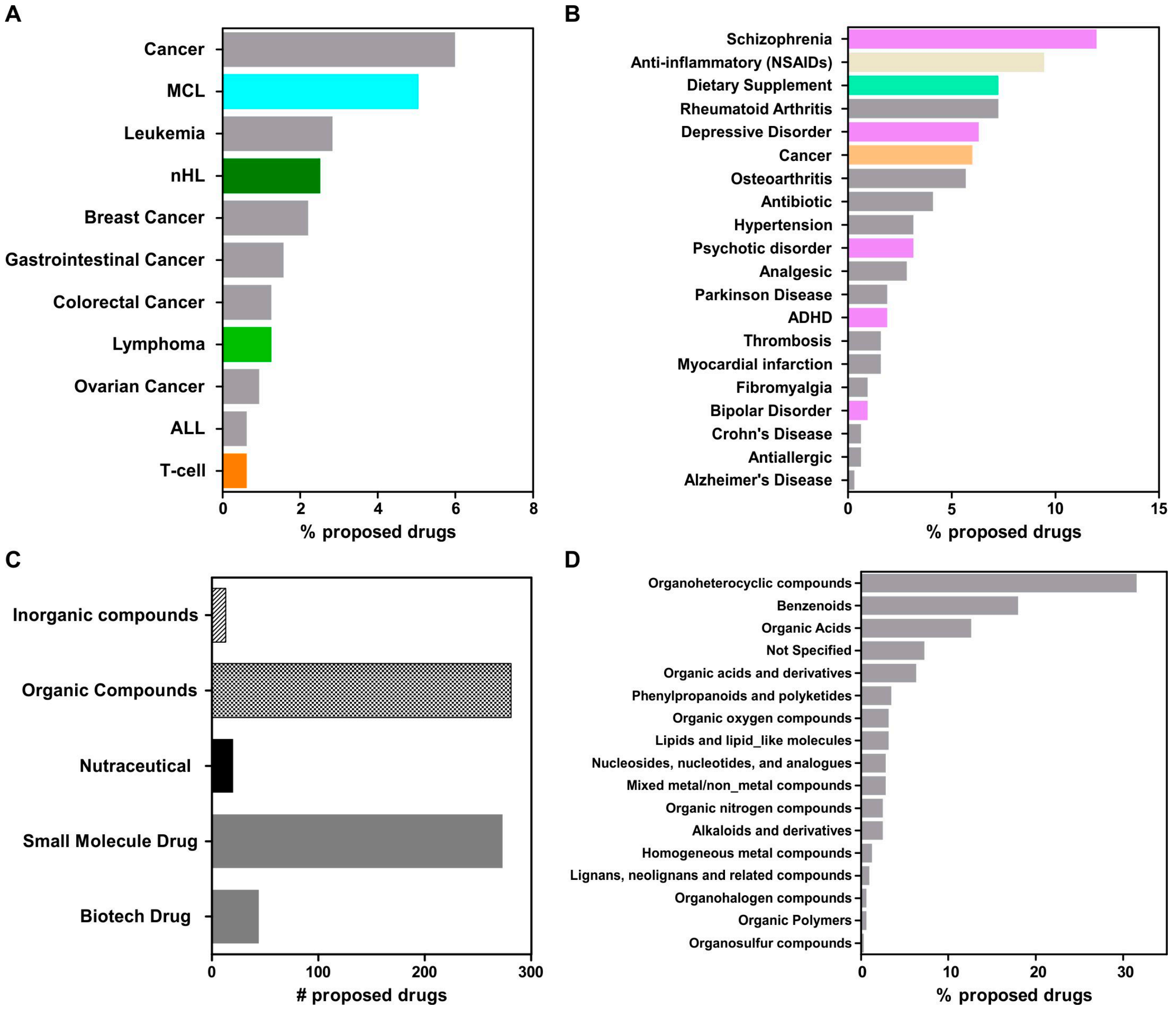

Breaking down the proposed drugs into different categories, based on compound information collected from DrugBank database: **A:** Cancer Type indication **B:** Disease Indication **C:** Compound Type **D:** Compound kingdom. Most of the proposed drugs are small molecules and organic compounds (273 and 281 drugs correspondingly). Three drugs are already used for the treatment of MCL (Ibrutinib, Acalabrutinib and Zanubrutinib), another 13 compounds (~4%) are at clinical trials for the treatment of relapsed and refractory MCL cases. Large part of the proposed drugs (77 drugs, ~24%) are primarily applied for the treatment mental illnesses: schizophrenia, depressive disorder, psychotic disorder, ADHD, and bipolar disorder. Other indications of the proposed drugs include rheumatoid arthritis, osteoarthritis, Parkinson's disease, thrombosis, myocardial infarction Crohn's disease and Alzheimer's disease. Drugbank also assigns compounds into kingdoms, based on chemical structure features. The most frequent are organoheterocyclic compounds, benzenoids and organic acids in our list.

<u>Supplementary Figure S10:</u> Validation of compound pair prediction using known MCL regiments.

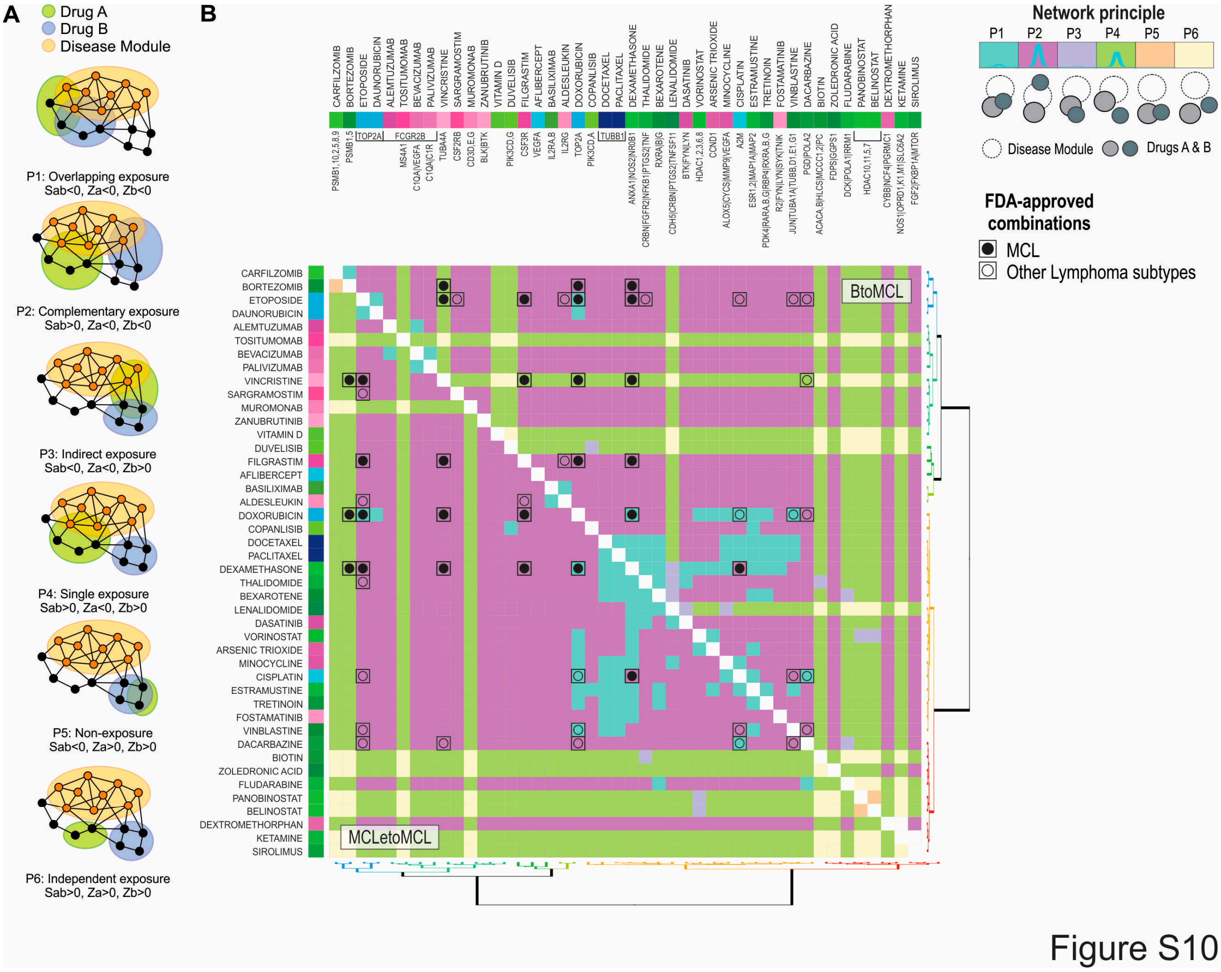

A: Cheng et al.[6] method classifies compound pairs into six network principles, based on three network proximities measured within the human PPIN (separation S_{ab} between two compounds A and B and the z-score distances Z_a and Z_b of the respective compounds from the disease module). Negative or positive S_{ab} values determine the topological overlap or separation of the targets of the two compounds correspondingly. The same way Z values define the ovelap between the targets of each compound with the disease module. As effective compound combinations are defined those classified into the second network principle (the two compounds overlap the disease module, but they are targeting different proteins). **B:** Validation of Cheng et al method using a set of 45 compounds included in previously described treatment cocktails of MCL (Table S1B). The top 100 DEGs of BtoMCL and MCLetoMCL comparisons were used as disease modules (Table S4). Split heatmap depicts the network principle of BtoMCL (bottom left half) and MCLetoMCL (top right half). Twenty-eight compound pairs are highlighted with filled and clear dots, these are agents used in multi-drug therapies applied in MCL or different lymphoma types (Table S1B).

<u>Supplementary Figure S11:</u> Predicted drug combinations at different MCL stages.

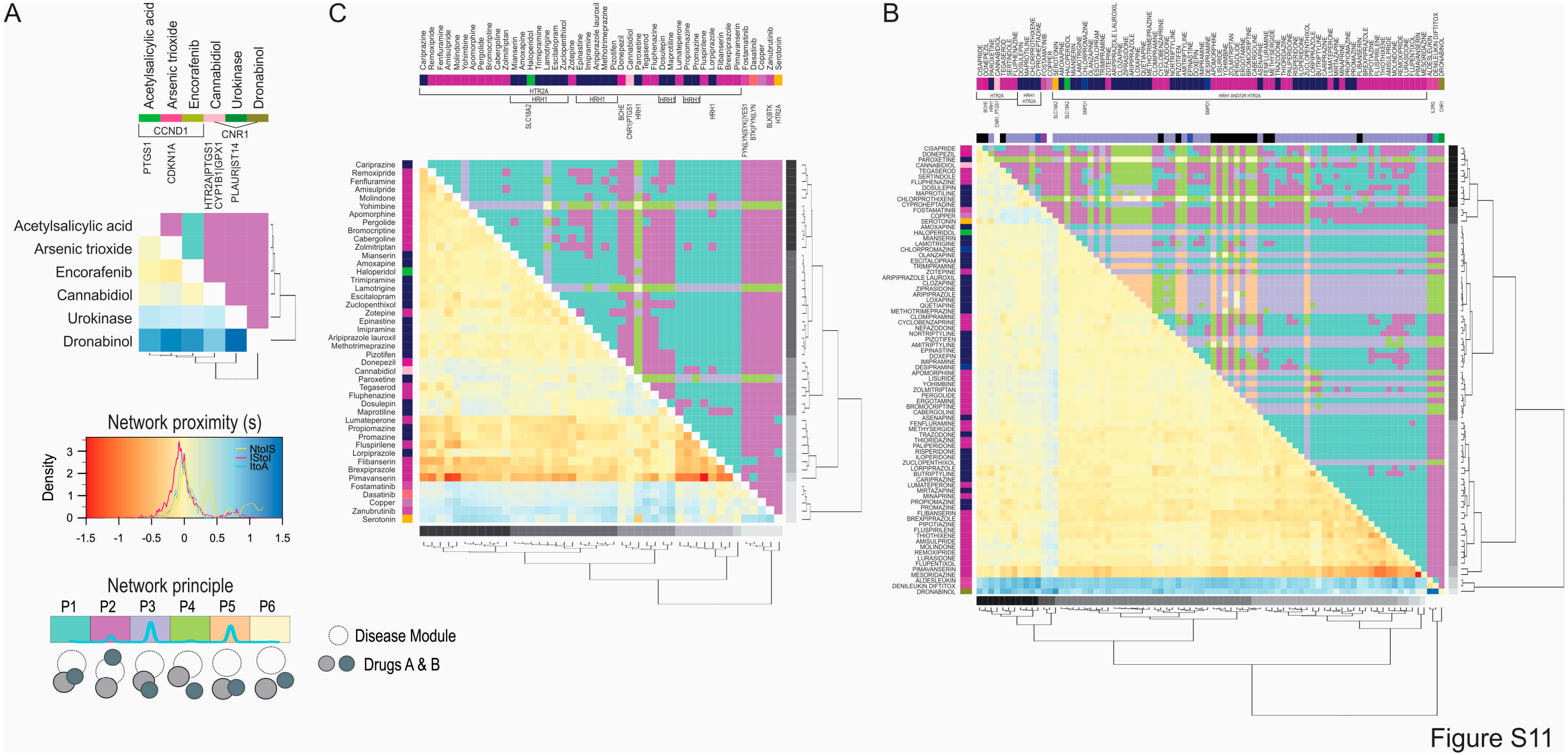

Network-based stratification of drug combinations for the treatment of MCL. Dual heatmaps present both the network proximity of drug-drug pairs (*s*; lower half) and the network principle of the drug pair given a disease module (upper half). Three drug combinations analyses are summarized here, each differing in the selected disease module, and the list of proposed drugs tested: **A.** NtoIS **B.** IStoI and **C.** ItoA. In each analysis, the disease module is defined as the set of top 100 DEGs identified in the respective sample comparison, while only the pairs of the respective proposed drugs are tested (6, 44 and 80 drugs respectively). Drugs with less than two protein targets were excluded from the network proximity estimation approach. Hierarchical clustering of drugs is based on

the separation (s) values, and clusters are colored in grey shades (rightmost hierarchical tree). Drugs names are colored based on the gene targets (top color bars).

Supplemental Tables

Supplementary Table S1: Short overview of existing therapies, repurposed drugs and deregulated biological pathways in MCL

A. FDA-approved drugs and existing therapies for different Lymphoma subtypes **B.** FDA-approved therapies originally used for other diseases that have been tested on lymphoma **C.** Known pathways affected in different lymphoma subtypes. D. Relevant publications.

Supplementary Table S2: SVM models trained for the separation of MCL stages.

We trained SVM models to separate N, IS, I, A stages; classical stage was left out to be predicted by the models. The models were trained using bootstrapping method (number of iterations: 100), leaving out $\frac{test}{total} = 25\%$ of each group of samples as a test set. The table summarizes SMV models trained with polynomial or radial basis kernel and the predictions for the four samples of the classical stage (GSM747375-8). Classical stage sample GSM747378 was not systematically predicted to the same class, therefore was removed from further analysis, whereas the reminder three samples were incorporated in the A stage. (N: Benign lymphadenitis, IS: *In Situ*, I: Intermediate, A: Aggressive)

Supplementary Table S3: Predicting Classical Stage samples using linear SVM classifier.

Class prediction of the four Classical stage samples GSM747375-8, using the best performing SMV model that is the linear SVM classifier (see Table S2). In

Supplementary Table S4: Catalogue of the top differentially expressed transcripts.

The combined list of the top 100 DEGs with p-value lower than 5% of all comparisons tested in the current study (NtoIS, IStoI, ItoA, NtoI, NtoA, HtoMCL, HtoP). The gene names and the respective Uniprot accession numbers (if applicable) are provided, along with the log fold change values and the adjusted p-values provided by the limma R package. The last column contains the list of comparisons, where each gene was identified as being differentially expressed.

Supplementary Table S5: Enriched pathways in MCL.

Comprehensive catalogue of the enriched pathways identified by: **A.** pathfindR tool (Ulgen E, Ozisik O, Sezerman OU (2018) pathfindR: An R Package for Pathway Enrichment Analysis Utilizing Active Subnetworks. bioRxiv) and **B.** PaintOmics 3.0 [72]. **C.** list of selected pathways that are depicted in figure's 2B heatmap. The full names and the KEGG identifiers are provided for every pathway. In the case of pathfindR the output includes the fold enrichment values (FE), the p-values, the number of occurrences (the number of network searches that a pathway was found to be enriched) and the lists of up-/down- regulated genes, whereas from PaintOmics a combined Fisher's p-value and the number of genes is available.

Supplementary Table S6 Proposed drug for MCL treatment.

Combined list of proposed drugs, all comparisons accounted: **A.** drug-gene relations and **B.** drugs. Every pair of drug-gene is described by the type of the drug's action on the gene, the list of comparisons where the gene was found to be significantly differentially expressed and the direction of regulation (up or down). The catalogue of top drugs includes the targeted genes per drug, as well as the respective comparisons, actions, and biological pathways. Drug-gene screening was performed, and certain pairs were excluded based on drug's action (see supplementary materials and methods).

Supplementary Table S7 Aligned common modules discovered with spectral clustering.

Implementation a spectral clustering approximation[70], for the alignment of functional modules between pairs of co-expression networks. **A.** Parameters used in each spectral clustering **B.** Common functional modules that were identified. Co-expression adjacency matrices were filtered, and weak relations (5% quartile) were flattened prior to spectral clustering. Co-expression networks of different stages were compared in the same manner as in the comparative analysis. K-means is extremely sensitive to cluster center initialization; therefore, we performed 100 repetitions, and measured the average and maximum number of modules. Hierarchical clustering is more robust than

k-means, in higher dimensional spaces (larger k values such as in the CtoI and ItoA comparisons). In some cases, the aligned modules contained unconnected nodes; these were manually excluded. The starting and remaining number of nodes, as well as the gene names, are provided for every module.

References

1. Varma, S. Blind estimation and correction of microarray batch effect. *PLoS One* **15**, e0231446 (2020).
2. Zhang, S. Comparisons of gene coexpression network modules in breast cancer and ovarian cancer. *BMC Syst Biol* **12**, 8 (2018).
3. Wishart, D. S. *et al.* DrugBank 5.0: a major update to the DrugBank database for 2018. *Nucleic Acids Res* **46**, D1074–D1082 (2018).
4. Shannon, P. *et al.* Cytoscape: a software environment for integrated models of biomolecular interaction networks. *Genome Res* **13**, 2498–2504 (2003).
5. Kanehisa, M., Furumichi, M., Tanabe, M., Sato, Y. & Morishima, K. KEGG: new perspectives on genomes, pathways, diseases and drugs. *Nucleic Acids Res* **45**, D353–D361 (2017).
6. Cheng, F., Kovács, I. A. & Barabási, A.-L. Network-based prediction of drug combinations. *Nat. Commun.* **10**, 1197 (2019).
7. Liu, Y. *et al.* DCDB 2.0: a major update of the drug combination database. *Database (Oxford).* **2014**, bau124 (2014).
8. Polakis, P. Wnt signaling in cancer. *Cold Spring Harb. Perspect. Biol.* **4**, (2012).
9. Kimura, Y. *et al.* The Wnt signaling pathway and mitotic regulators in the initiation and evolution of mantle cell lymphoma: Gene expression analysis. *Int J Oncol* **43**, 457–468 (2013).
10. Zhan, T., Rindtorff, N. & Boutros, M. Wnt signaling in cancer. *Oncogene* **36**, 1461–1473 (2017).
11. Kafri, P. *et al.* Quantifying β-catenin subcellular dynamics and cyclin D1 mRNA transcription during Wnt signaling in single living cells. *Elife* **5**, e16748 (2016).
12. Sebio, A. & Lenz, H. J. Molecular Pathways: Hippo Signaling, a Critical Tumor Suppressor. *Clin Cancer Res* **21**, 5002–5007 (2015).
13. Bae, J. S., Kim, S. M. & Lee, H. The Hippo signaling pathway provides novel anti-cancer drug targets. *Oncotarget* **8**, 16084–16098 (2017).
14. Menke, A. *et al.* Down-regulation of E-cadherin gene expression by collagen type I and type III in pancreatic cancer cell lines. *Cancer Res* **61**, 3508–3517 (2001).
15. Shargh, S. A. *et al.* Downregulation of E-cadherin expression in breast cancer by promoter hypermethylation and its relation with progression and prognosis of tumor. *Med Oncol* **31**, 250 (2014).
16. Li, X. *et al.* Snail induction is an early response to Gli1 that determines the efficiency of epithelial transformation. *Oncogene* **25**, 609–621 (2006).
17. Chang, Y. *et al.* Activated hippo signal pathway inhibits cell proliferation and promotes apoptosis in NK/T cell lymphoma cells. *Cancer Med.* **8**, 3892–3904 (2019).
18. Zanconato, F., Cordenonsi, M. & Piccolo, S. YAP and TAZ: a signalling hub of the tumour microenvironment. *Nat. Rev. Cancer* **19**, 454–464 (2019).
19. Xiao, C. *et al.* Hedgehog signaling regulates E-cadherin expression for the maintenance of the actin cytoskeleton and tight junctions. *Am J Physiol Gastrointest Liver Physiol* **299**, G1252-65 (2010).
20. Katoh, Y. & Katoh, M. Hedgehog signaling, epithelial-to-mesenchymal transition and miRNA (review). *Int. J. Mol. Med.* **22**, 271–275 (2008).
21. Venerando, A. *et al.* Isoform specific phosphorylation of p53 by protein kinase CK1. *Cell. Mol. Life Sci.* **67**, 1105–1118 (2010).
22. Grozav, A. G. *et al.* Casein kinase I delta/epsilon phosphorylates topoisomerase IIalpha at serine-1106 and

modulates DNA cleavage activity. *Nucleic Acids Res.* **37**, 382–392 (2009).

23. Schittek, B. & Sinnberg, T. Biological functions of casein kinase 1 isoforms and putative roles in tumorigenesis. *Mol. Cancer* **13**, 231 (2014).

24. Inuzuka, H. *et al.* Phosphorylation by casein kinase I promotes the turnover of the Mdm2 oncoprotein via the SCF(beta-TRCP) ubiquitin ligase. *Cancer Cell* **18**, 147–159 (2010).

25. Merolle, M. I., Ahmed, M., Nomie, K. & Wang, M. L. The B cell receptor signaling pathway in mantle cell lymphoma. *Oncotarget* **9**, 25332–25341 (2018).

26. Rodgers, T. D. & Barr, P. M. Pitfalls of Combining Novel Agents in Lymphoma. *Curr. Treat. Options Oncol.* **19**, 35 (2018).

27. Younes, A. *et al.* The landscape of new drugs in lymphoma. *Nat Rev Clin Oncol* **14**, 335–346 (2017).

28. Guinamard, R., Okigaki, M., Schlessinger, J. & Ravetch, J. V. Absence of marginal zone B cells in Pyk-2-deficient mice defines their role in the humoral response. *Nat. Immunol.* **1**, 31–36 (2000).

29. Waghela, B. N. *et al.* AGE-RAGE synergy influences programmed cell death signaling to promote cancer. *Mol. Cell. Biochem.* **476**, 585–598 (2021).

30. Soman, S. *et al.* A multicellular signal transduction network of AGE/RAGE signaling. *J. Cell Commun. Signal.* **7**, 19–23 (2013).

31. Logsdon, C. D., Fuentes, M. K., Huang, E. H. & Arumugam, T. RAGE and RAGE ligands in cancer. *Curr. Mol. Med.* **7**, 777–789 (2007).

32. Zhao, W.-L. *et al.* Vascular endothelial growth factor-A is expressed both on lymphoma cells and endothelial cells in angioimmunoblastic T-cell lymphoma and related to lymphoma progression. *Lab. Invest.* **84**, 1512–1519 (2004).

33. Dvorak, H. F. Rous-Whipple Award Lecture. How tumors make bad blood vessels and stroma. *The American journal of pathology* vol. 162 1747–1757 (2003).

34. Jiang, L. & Li, N. B-cell non-Hodgkin lymphoma: importance of angiogenesis and antiangiogenic therapy. *Angiogenesis* **23**, 515–529 (2020).

35. Potti, A., Ganti, A. K., Kargas, S. & Koch, M. Immunohistochemical detection of C-kit (CD117) and vascular endothelial growth factor (VEGF) overexpression in mantle cell lymphoma. *Anticancer Res.* **22**, 2899–2901 (2002).

36. Heider, U. *et al.* Histone deacetylase inhibitors reduce VEGF production and induce growth suppression and apoptosis in human mantle cell lymphoma. *Eur. J. Haematol.* **76**, 42–50 (2006).

37. Zhang, L.-H., Schafer, P. H., Muller, G., Stirling, D. & Bartlett, B. Direct Inhibitory Effects of Lenalidomide on the Proliferation and VEGF Production of Non-Hodgkin Lymphoma Cells Are Associated with Increased SPARC Expression. *Blood* **112**, 2612 (2008).

38. Podhajcer, O. L. *et al.* The role of the matricellular protein SPARC in the dynamic interaction between the tumor and the host. *Cancer Metastasis Rev.* **27**, 523–537 (2008).

39. Clark, C. J. & Sage, E. H. A prototypic matricellular protein in the tumor microenvironment--where there's SPARC, there's fire. *J. Cell. Biochem.* **104**, 721–732 (2008).

40. Nagaraju, G. P., Dontula, R., El-Rayes, B. F. & Lakka, S. S. Molecular mechanisms underlying the divergent roles of SPARC in human carcinogenesis. *Carcinogenesis* **35**, 967–973 (2014).

41. Ahir, B. K., Elias, N. M. & Lakka, S. S. SPARC overexpression alters microRNA expression profiles involved in tumor progression. *Genes Cancer* **8**, 453–471 (2017).

42. Nayak, A. P., Kapur, A., Barroilhet, L. & Patankar, M. S. Oxidative Phosphorylation: A Target for Novel

Therapeutic Strategies Against Ovarian Cancer. *Cancers (Basel).* **10**, 337 (2018).

43. Zhang, L. *et al.* Metabolic reprogramming toward oxidative phosphorylation identifies a therapeutic target for mantle cell lymphoma. *Sci. Transl. Med.* **11**, (2019).

44. Grillone, K. *et al.* Non-coding RNAs in cancer: platforms and strategies for investigating the genomic "dark matter". *J. Exp. Clin. Cancer Res.* **39**, 117 (2020).

45. Liu, Y. *et al.* Noncoding RNAs regulate alternative splicing in Cancer. *J. Exp. Clin. Cancer Res.* **40**, 11 (2021).

46. Mu, Q. *et al.* Imp2 regulates GBM progression by activating IGF2/PI3K/Akt pathway. *Cancer Biol. Ther.* **16**, 623–633 (2015).

47. Kessler, S. M. *et al.* IMP2/IGF2BP2 expression, but not IMP1 and IMP3, predicts poor outcome in patients and high tumor growth rate in xenograft models of gallbladder cancer. *Oncotarget* **8**, 89736–89745 (2017).

48. Dai, N. *et al.* IGF2 mRNA binding protein-2 is a tumor promoter that drives cancer proliferation through its client mRNAs IGF2 and HMGA1. *Elife* **6**, (2017).

49. Gao, T., Liu, X., He, B., Pan, Y. & Wang, S. Long non-coding RNA 91H regulates IGF2 expression by interacting with IGF2BP2 and promotes tumorigenesis in colorectal cancer. *Artif. cells, nanomedicine, Biotechnol.* **48**, 664–671 (2020).

50. Pradella, D. *et al.* A ligand-insensitive UNC5B splicing isoform regulates angiogenesis by promoting apoptosis. *Nat. Commun.* **12**, 4872 (2021).

51. Wang, H., Su, H. & Tan, Y. UNC5B-AS1 promoted ovarian cancer progression by regulating the H3K27me on NDRG2 via EZH2. *Cell Biol. Int.* **44**, 1028–1036 (2020).

52. Wang, Y. *et al.* The lncRNA UNC5B-AS1 promotes proliferation, migration, and invasion in papillary thyroid cancer cell lines. *Hum. Cell* **32**, 334–342 (2019).

53. Zhang, M. *et al.* LncRNA CBR3-AS1 regulates of breast cancer drug sensitivity as a competing endogenous RNA through the JNK1/MEK4-mediated MAPK signal pathway. *J. Exp. Clin. Cancer Res.* **40**, 41 (2021).

54. Wang, Y., Zhao, Y., Zhang, X., Zhang, A. & Ma, J. Long noncoding RNA LBX2-AS1 drives the progression of hepatocellular carcinoma by sponging microRNA-384 and thereby positively regulating IRS1 expression. *Pathol. Res. Pract.* **216**, 152903 (2020).

55. Li, Z.-B., Chu, H.-T., Jia, M. & Li, L. Long noncoding RNA LINC01139 promotes the progression of hepatocellular carcinoma by upregulating MYBL2 via competitively binding to miR-30 family. *Biochem. Biophys. Res. Commun.* **525**, 581–588 (2020).

56. Wu, L. *et al.* Long non-coding RNA LINC01003 suppresses the development of multiple myeloma by targeting miR-33a-5p/PIM1 axis. *Leuk. Res.* **106**, 106565 (2021).

57. Yu, H. *et al.* Identification and Validation of Long Noncoding RNA Biomarkers in Human Non–Small-Cell Lung Carcinomas. *J. Thorac. Oncol.* **10**, 645–654 (2015).

58. Nie, K. *et al.* Identification of a 14-lncRNA Signature and Construction of a Prognostic Nomogram Predicting Overall Survival of Gastric Cancer. *DNA Cell Biol.* **39**, 1532–1544 (2020).

59. Ballou, Y. *et al.* 5-HT serotonin receptors modulate mitogenic signaling and impact tumor cell viability. *Mol. Clin. Oncol.* **9**, 243–254 (2018).

60. Chubak, J., Boudreau, D. M., Rulyak, S. J. & Mandelson, M. T. Colorectal cancer risk in relation to antidepressant medication use. *Int. J. cancer* **128**, 227–232 (2011).

61. Ochs, M. J., Suess, B. & Steinhilber, D. 5-lipoxygenase mRNA and protein isoforms. *Basic Clin. Pharmacol. Toxicol.* **114**, 78–82 (2014).

62. Ponzetti, M. *et al.* Non-conventional role of haemoglobin beta in breast malignancy. *Br J Cancer* **117**, 994–1006 (2017).

63. Capulli, M. *et al.* Increased expression of a set of genes enriched in oxygen binding function discloses a predisposition of breast cancer bone metastases to generate metastasis spread in multiple organs. *J Bone Min. Res* **27**, 2387–2398 (2012).

64. Gallo, D., De Stefano, I., Grazia Prisco, M., Scambia, G. & Ferrandina, G. Estrogen receptor beta in cancer: an attractive target for therapy. *Curr Pharm Des* **18**, 2734–2757 (2012).

65. Ringleb, J. *et al.* Apoptotic Cancer Cells Suppress 5-Lipoxygenase in Tumor-Associated Macrophages. *J Immunol* **200**, 857–868 (2018).

66. Li, X. Y. *et al.* The antitumor effects of arsenic trioxide in mantle cell lymphoma via targeting Wnt/betacatenin pathway and DNA methyltransferase-1. *Oncol Rep* **38**, 3114–3120 (2017).

67. Shaw, V., Srivastava, S. & Srivastava, S. K. Repurposing antipsychotics of the diphenylbutylpiperidine class for cancer therapy. *Semin. Cancer Biol.* **68**, 75–83 (2021).

68. Tuan, N. M. & Lee, C. H. Penfluridol as a Candidate of Drug Repurposing for Anticancer Agent. *Molecules* **24**, (2019).

69. Ardalani, H., Avan, A. & Ghayour-Mobarhan, M. Podophyllotoxin: a novel potential natural anticancer agent. *Avicenna J. phytomedicine* **7**, 285–294 (2017).

70. Zhang, S., Zhao, H. & Ng, M. K. Functional Module Analysis for Gene Coexpression Networks with Network Integration. *IEEE/ACM Trans Comput Biol Bioinform* **12**, 1146–1160 (2015).

71. Meyer, P. E., Lafitte, F. & Bontempi, G. minet: A R/Bioconductor Package for Inferring Large Transcriptional Networks Using Mutual Information. *BMC Bioinformatics* **9**, 461 (2008).

72. Garcia-Alcalde, F., Garcia-Lopez, F., Dopazo, J. & Conesa, A. Paintomics: a web based tool for the joint visualization of transcriptomics and metabolomics data. *Bioinformatics* **27**, 137–139 (2011).
